# Meteorologically Driven Changes in Future Global Air Quality: Physical and Monetized Impacts

**DOI:** 10.1021/acs.est.5c14713

**Published:** 2026-07-02

**Authors:** Erin E. McDuffie, Lee T. Murray, Sebastian D. Eastham, Melanie Jackson, Marcus C. Sarofim, William Raich, Richard Burnett, Jim Anderton, Henry Roman, James E. Neumann, Simone Tilmes, Kwesi A. Quagraine, Neal Fann

**Affiliations:** † EPA Office of Atmospheric Protection, Washington, D.C. 20460, United States; ‡ Department of Earth and Environmental Sciences, 6927 University of Rochester, Rochester, New York 14627, United States; § Department of Aeronautics, Imperial College London, London SW7 2AZ, United Kingdom; ∥ 53789 Industrial Economics, Incorporated, Cambridge, Massachusetts 02140, United States; ⊥ 164 Fanshaw Avenue, Ottawa, Ontario K1H6C9, Canada; # NSF National Center for Atmospheric Research, Boulder, Colorado 80305, United States; ∇ Harvard University, Cambridge, Massachusetts 02138, United States

**Keywords:** air pollution, mortality, meteorological projections, cost, benefit

## Abstract

Exposure to ambient air pollution, including ozone and fine particulate matter (PM_2.5_), is the world’s leading environmental health risk factor. Estimating how this burden may change in the future depends on projecting population growth and age structure as well as understanding how future meteorological changes may impact the production and removal of pollutants from the atmosphere. The net impact of these factors on a global scale has not been well-characterized. Here, we leverage recent meteorology, exposure, and mortality output from general circulation, atmospheric chemistry, and health impact models to isolate how changes in meteorology and populations will impact future global air-pollution-related mortality and the associated monetized impacts by the degree of global temperature change. In contrast to previous studies, we estimate that changes in meteorologically driven air pollution, in the absence of pollutant precursor emission changes, will result in 180 000 fewer deaths annually by 2100 relative to current levels, an annual monetized benefit of $7.3 trillion. Reductions are driven by decreases in PM_2.5_-attributable mortality in populated regions but are substantially offset by global increases in ozone-related mortality. We also highlight striking regional differences in the sign of net pollutant impacts by 2100, with net pollution decreases in the Northern Hemisphere driven by reductions in nitrate aerosol, while increases in both ozone and organic aerosol at higher temperatures lead to net increases in pollutant impacts in the Southern Hemisphere. Lastly, we assess sensitivities of these results to meteorological projections, health impact functions, and 10 000 future warming scenarios.

## Graphical abstract

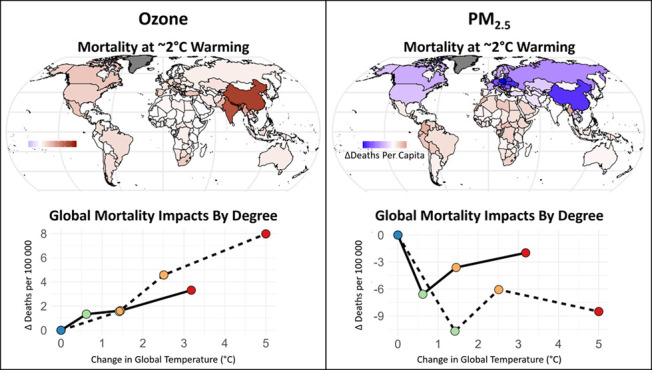

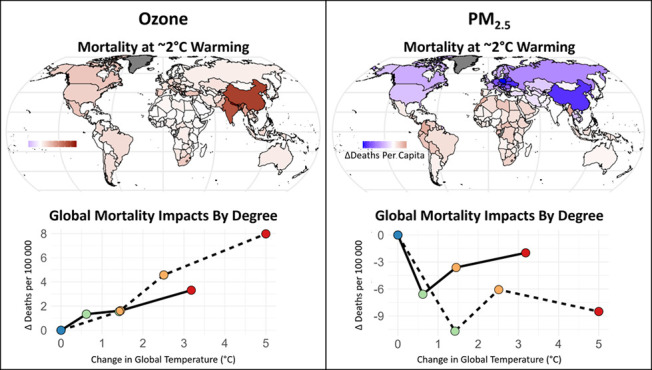

## Introduction

Long-term exposure to ambient air pollution, including ground-level ozone (O_3_) and fine particulate matter (PM_2.5_) currently results in over 5 million deaths worldwide each year,^1,2^ with the largest health burden found in populated regions throughout Asia.^3^ Future exposure to air pollution will depend on the complex production and loss mechanisms of O_3_ and PM_2.5_ in the atmosphere, which are distinct from each other and highly variable in time and space. Understanding how this burden and the associated monetized impacts are expected to change in the future has important consequences for understanding future societal risks and for informing future air quality planning.

Ozone is formed from the chemical reaction of nitrogen oxides (NO_*x*_) and volatile organic compounds or carbon monoxide in the presence of sunlight. In contrast, PM_2.5_ has multiple direct sources, including combustion, as well as through the condensation or thermodynamic partitioning of gases to the aerosol phase, which are emitted from a combination of energy, industrial, agricultural, and natural sources. In terms of loss, O_3_ is primarily removed from the atmosphere by photolysis in the presence of water vapor,^4^ and secondarily through dry deposition and uptake by vegetation,^5^ while PM_2.5_ is efficiently removed by precipitation.^6,7^ Therefore, net concentrations of these air pollutants in the future and the associated health and monetized impacts will depend on a complex combination of local emission sources, regional transport patterns, land cover, and other meteorological conditions such as temperature and precipitation patterns,^8^ as well as the size and age distribution of the most highly exposed populations.^9^

Numerous previous studies have used a combination of general circulation models and atmospheric chemical transport models to estimate future changes in O_3_ and PM_2.5_ concentrations.^8,10–21^ These studies have provided valuable insight into a range of possible futures associated with various emission scenarios. However, as O_3_ and PM_2.5_ are highly sensitive to specific scenario emission inputs,^17^ it is also valuable to isolate the changes in PM_2.5_ and O_3_ concentrations that are expected to occur only from changes in meteorological drivers and the associated natural feedbacks, not anthropogenic emissions. This isolation enables us to assess the potential sign and magnitude of the PM_2.5_ and O_3_ health burden to future populations, regardless of underlying assumptions about the likelihood of future temperatures or anthropogenic emissions.

Many fewer studies have estimated these isolated impacts of changes in meteorology (e.g., temperature and precipitation) on both air pollutant concentrations and the associated health burden,^15,22–28^ and none have monetized these impacts of both pollutants on a global scale. While these studies generally agree that future O_3_-related mortality will increase in populated regions like the U.S. and China, they disagree on both the magnitude and direction of the predicted changes in PM_2.5_-related mortality. Direct comparisons between these studies, however, are complicated by their use of different atmospheric models, assumptions about PM_2.5_ components, future population sizes and age distributions, and different health impact functions. These discrepancies lead to uncertainties in whether changes in meteorological conditions will result in a net damage or benefit to future populations relative to current conditions.

This work builds off these previous studies by leveraging the most recent advances in models of general circulation, atmospheric chemistry, and health impacts to assess the sensitivity of PM_2.5_- and O_3_-related mortality to changes in future meteorology at the global scale. Using these results to develop by degree impact functions for each country and pollutant, we model 10 000 probabilistic scenarios of future temperature, population, and GDP, to estimate the annual physical and monetized impacts in each country by the end of the century. This is the first study to estimate the net global monetized impacts of meteorologically driven changes in air pollution, which reveals regional variability that helps to better understand the potential risks and benefits associated with future changes in air pollution.

## Materials and Methods

This analysis uses a multistep analytical approach (Figure 1) to estimate the change in global air pollution-related deaths associated with future changes in meteorology and population. First, two General Circulation Models (GCMs) from the sixth Coupled Model Intercomparison Project (CMIP6) provide the meteorological parameters from a recent historical period (2005–2014) and three future (2090–2099) scenarios. Second, we format these parameters for input into 8 (2 baseline, 6 future) individual simulations using the GEOS-Chem atmospheric chemistry transport model (CTM). Third, resulting global gridded (2° × 2.5° resolution) O_3_ and PM_2.5_ concentrations are averaged over each simulation period (2005–2014 or 2090–2099), bias-corrected, and downscaled to 0.5° × 0.5° for input into the EPA’s global Environmental Benefits Mapping and Analysis Program (BenMAP) webtool. BenMAP estimates the annual mortality in each country associated with each pollutant and exposure level. Lastly, we combine annual mortality results from each future scenario with global temperature changes from each GCM to develop by degree impact functions and use these to estimate the change in the physical and monetized impacts of meteorologically driven changes in air pollution in each country by the end of the century_._ Details of each step are described in the following sections.

**Figure 1:**
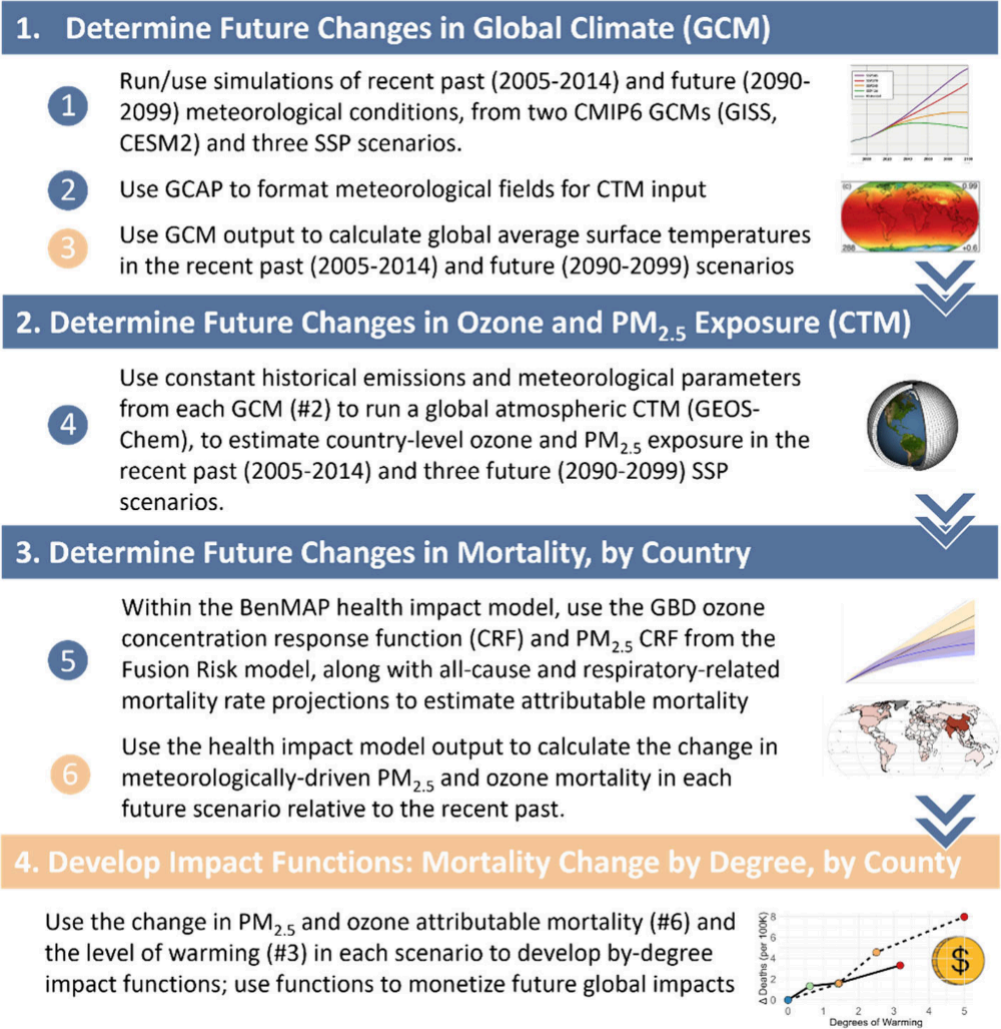
Schematic of analytical workflow. Blue, inputs and models; yellow, outputs used to develop impact functions.

### Future Changes in Meteorology

We first leverage projections of future meteorological conditions from two GCMs from the most recent CMIP6 ^29^ (Table 1). The first is NASA’s Goddard Institute for Space Studies (GISS) ModelE2.1, run for CMIP6, with meteorological parameters previously archived and formatted using version 2 of the GCAP framework.^23^ The second is the Community Earth System Model 2 ^30^ (hereafter CESM), which uses the Community Atmosphere Model version 6 (CAM6),^31^ and was used by the National Center for Atmospheric Research to generate output for CMIP6. The Global Change and Air Pollution (GCAP) model framework provides an offline one-way coupling between the GISS GCM and the GEOS-Chem CTM^23^ and was adapted for this study to format and utilize output from CESM. To archive the specific meteorological variables from CESM that are required for the GCAP framework, we reran the CESM model for this analysis using the same CMIP6 ^30^ model configuration and emissions, but taking archived CMIP6 initial conditions and sea surface temperatures as a boundary condition for CAM6 in place of an online ocean model. Meteorological parameters from both the GISS and CESM models were archived at a 3 h temporal resolution for 3D fields and 1 h for 2D fields. The resulting meteorological parameters were formatted for input into the GEOS-Chem CTM using the GCAP framework. We use GCM output for the recent historical period (2005–2014), as well as for the 2090s decade (2090–2099), driven with emissions from three SSP scenarios, which represent a range of future warming levels (Supplemental Text S1). We selected the 2090s decade to provide the largest possible range of future global temperature changes relative to the baseline to support the development of the by degree impact functions. Table S1 shows the specific CMIP6 GCM scenarios used in this analysis, along with a comparison of the global average temperatures across each scenario and GCM.

**Table 1:** GEOS-Chem CTM Air Quality Simulation Inputs and Output Air Quality Results

scenario number^a^	anthropogenic emissions	meteorological inputs^b^	biogenic NMVOCs^c^ (Tg of C/year)	MDA8O_3_ (ppb) (min, mean, max change)^d^	average PM_2.5_ (μg/m^3^) (min, mean, max change)^e^
1, 2	2005–2014 average:	2005–2014	770 (GISS)	37.1 (CESM)	16.3 (CESM)
	SO_2_: 110 Tg/year		700 (CESM)	38.8 (GISS)	16.3 (GISS)
	NH_3_: 58 Tg/year				
	NO: 75 Tg/year				
	CO: 610 Tg/year				
	NMVOC: 120 Tg of C/year				
	OC + BC: 26 Tg/year				
3	2005–2014	GISS 2090–2099 SSP1-2.6	840	38.7 (−5.4, −0.1, 7.8)	16.1 (−14.7, −0.2, 1.6)
4	2005–2014	GISS 2090–2099 SSP2-4.5	960	38.8 (−5.3, 0, 6.1)	16.2 (−12.4, −0.1, 1.9)
5	2005–2014	GISS 2090–2099 SSP5-8.5	1220	39.3 (−6.4, 0.5, 11.7)	16.4 (−13.4, 0.1, 4.3)
6	2005–2014	CESM 2090–2099 SSP1-2.6	890	35.6 (−4.7, −1.5, 9.5)	15.9 (−17.1, −0.5, 3.7)
7	2005–2014	CESM 2090–2099 SSP2-4.5	1030	38.7 (−4, 1.6, 12)	16.0 (−15.4, −0.3, 3.1)
8	2005–2014	CESM 2090–2099 SSP5-8.5	1480	40.1 (−7.7, 3, 23.3)	16.4 (−18.2, 0.1, 8.2)

a Each simulation has a duration of 10 years.

b Three of the seven SSP scenarios were chosen to reflect the broadest possible range of future conditions, ranging from low warming and pollutant precursor emissions (e.g., SSP1-2.6) to a higher warming and emissions scenario (SSP5-8.5). The choice of scenarios used in this work does not provide an endorsement for or reflect the likelihood of a specific scenario outcome.

c Emissions of biogenic VOCs are estimated during model runtime, given the input meteorological conditions.

d Bias-corrected, land average ozone season MDA8O_3_ (ppb).

e Bias-corrected, annual, land average average PM_2.5_ concentrations.

### Future Changes in Global Ozone and PM_2.5_ Exposure

The formatted meteorological parameters from each scenario were then input into the 3D global CTM GEOS-Chem 13.4.1 ^32,33^ to simulate air pollutant concentrations in each scenario. In contrast to the GCM’s, the more detailed chemical mechanism provided by the CTM is intended to better capture future changes in air pollutant concentrations than the GCM output. We use the ‘classic’ GEOS-Chem configuration, where simulations are performed on a single computational node and on a rectilinear latitude-longitude grid. GEOS-Chem solves for the global evolution of atmospheric gases and aerosols over time using archived inputs of meteorological data, input global and regional emission inventories, and algorithms that represent physical and chemical atmospheric processes. As described previously,^23^ GEOS-Chem was run for a 10 year reference period (2005–2014), as well as for the 2090s decade (2090–2099), driven by precursor emissions and meteorological parameters of each GCM/SSP combination (Table 1). We do not include the impact of emissions from open burning, such as wildfires on concentrations of PM_2.5_ and O_3_, which can be considered an additive effect (as discussed in the Results and Discussion). The GEOS-Chem model setup is the same as that previously described by ref 23, but both the GISS- and CESM-driven GEOS-Chem simulations were conducted specifically for this analysis. The performance of GEOS-Chem driven by the GISS meteorology versus *in situ* and satellite observations for the 2005–2014 CE period was extensively evaluated by ref 34. The same modeling system was applied to the CESM2 meteorology. Further details about the GEOS-Chem simulations are provided in Supporting Information Text S1. By only varying inputs of meteorological parameters and natural emission feedbacks [e.g., biogenic VOCs (BVOCs) and soil NO_*x*_], the changes in average concentrations of PM_2.5_ and O_3_ in the 2090s simulations relative to the reference period can be used to isolate meteorologically driven changes to future air quality. For the exposure metrics, we used the ozone season maximum daily 8 h average O_3_ (MDA8O_3_) concentrations and the annual average PM_2.5_ concentration (μg/m^3^), as calculated from the GEOS-Chem output for each 10 year simulation. All scenario results for O_3_ and PM_2.5_ are downscaled and bias corrected to recent observation-based products.^35,36^ While the resolution of the CTM cannot resolve all of the detailed transport, production, and loss processes occurring in population centers, we trade detail for global coverage and apply additional downscaling and bias correction to reduce the impact of model resolution at finer spatial scales and in regions with higher population density. Table 1 provides details about the inputs and select outputs from each GEOS-Chem simulation.

### Future Changes in Attributable Mortality by Country

We focus on respiratory-related mortality associated with MDA8O_3_, as well as mortality from noncommunicable diseases and lower respiratory infections (NCD + LRI) associated with long-term exposure to annual 24 h average concentrations of PM_2.5_. Exposure metrics used to quantify mortality associated with long-term exposure to O_3_ and PM_2.5_ are consistent with previous air quality health studies and EPA Integrated Assessments Reports.^37,38^ For O_3_, current epidemiological and toxicological research provides the strongest evidence for respiratory-related mortality (vs cardiovascular or other) resulting from long-term exposure to O_3_ ^38^ for ages 0–99. For PM_2.5_, there is strong epidemiologic evidence of all-cause mortality resulting from long-term exposure to ambient PM_2.5_ ^37^ for ages over 25. Due to the focus of this study on long-term changes in air pollution over multiple years, we did not consider additional mortality due to changes in short-term exposure to either air pollutant.

### Concentration Response Functions: Ozone

Consistent with previous similar analyses,^39,40^ we applied a chronic obstructive pulmonary disease (COPD) relative risk coefficient (beta) to all respiratory-related mortality in all countries for all ages (0–99). The relative risk coefficient of 1.06 per 10 ppb O_3_ exposure (95% CI: 1.03, 1.10) was derived from a meta-regression of five recent cohort studies in Canada, the United Kingdom, and the United States by the 2019 Global Burden of Disease^41^ (further details in Supporting Information Text S2). To facilitate the development of the reduced form functions, we assume a linear reduction (on the log-hazard ratio scale) in risk associated with any reduction in exposure (e.g., a theoretical minimum risk exposure level of 0 ppb), which may overestimate the health impacts in countries that are strongly influenced by marine air and have relatively low concentrations of MDAO_3_ but only impacts global total results by 1% (Figure S2, discussed further in Supporting Information Text S2).

### Concentration Response Functions: PM_2.5_

For PM_2.5_, we used a parameterized version of the Fusion risk model, applied to adults ages 25–99, which is a fusion of existing risk models that relate marginal changes in long-term PM_2.5_ exposure to marginal changes in relative risk of death.^42^ Underlying risk models include the Integrated Exposure Response Functions (IER), Global Exposure Mortality Model (GEMM), and log–linear model, the latter of which has been used in recent EPA health burden analyses.^37^ The Fusion model largely relies on the log–linear model relationship at low concentrations, typically representative of present day PM_2.5_ concentrations in the U.S., but uses functions with declining derivatives at higher exposure levels to ensure that relative risk estimates remain biologically plausible at the highest exposure levels. By both leveraging features and addressing limitations of these models, the Fusion model predicts relative risks that lie within the range of previous models, while better extrapolating risk above and below the exposure ranges observed in cohort studies.

For the parameterized version of the Fusion model relative risk (RR), we assume a reduction in risk associated with any reduction in exposure (e.g., down to a theoretical minimum risk exposure level of 0 μg/m^3^).^42^ The parameterized function is based on 1000 RR curves produced by the Fusion model,^42^ and approximates the mean using a series of arctangent functions. The standard error of the mean is calculated by equating the covariance of the 1000 predictions to the covariance of the mean prediction.^43^ The parameterization mimics the form of the log–linear model (β·*z*), where the function is β·*T*(*Q*), and *T*(*Q*) is a known transformation of *Q* indexed by a number of parameters (eqs 1 and 2).

$$\mathrm{RR}=e^{T\left(Q\right)\cdot \beta }$$ 1

$$T\left(Q\right)=\overset{4}{\underset{i=1}{\sum }}\tau_{i}\cdot \tan^{-1}\frac{Q}{\eta_{i}}$$ 2

*Q* refers to the PM_2.5_ concentration, while β, τ_*i*_, and η_*i*_ refer to parameters developed to approximate the RR function. See Supporting Information Text S2 for more information on the Fusion model and a table of the parameters used in this analysis.

### Background Mortality and Population

Estimates of NCD + LRI and respiratory-related baseline mortality for the 2090s decade were derived using the Resources for the Future Socioeconomics Projections (RFF-SPs). This data set contains 1000 probabilistic projections of country-level population and all-cause mortality from 2020 to 2300, stratified by age and sex.^44^ Since the RFF-SP data does not contain mortality estimates differentiated by cause of death, mortality projections from the International Futures Project (IFs) were used to adjust all-cause mortality rates by age, country, and year for specific causes of death.^45^ IFs data contains mortality counts for respiratory mortality and NCD + LRI mortality. This is the same approach for estimating future projections of cause-specific background mortality rates as previously described by ref 39, which shows, for example, that global average respiratory mortality rates are estimated to increase from roughly 50 to 150 deaths per 100 000 people between 2020 and 2100.

### BenMAP

Using the concentrations response functions (CRFs) and baseline mortality rates described above, we estimate PM_2.5_- and O_3_-attributable deaths using EPA’s BenMAP webtool. BenMAP estimates health impacts at the spatial scale of the air quality data (0.5° × 0.5°). Therefore, grid cells intersecting two or more countries are divided along these boundaries to ensure country-specific population and mortality data are properly assigned. As described elsewhere,^39^ country-level population data are downscaled to this same grid using the Gridded Population of the World,^46^ but BenMAP uses a single, country-specific baseline mortality rate for each grid cell within a single country. This analysis is the first application of a burden analysis of PM_2.5_ using the global version of BenMAP.

To quantify the annual change in the O_3_ health burden between each future scenario and the reference period, BenMAP uses a log–linear function based on the change in O_3_ concentrations (eq 3)­

$$y_{c,s}={\mathrm{incidence}}_{c}\cdot {\mathrm{population}}_{c}\cdot \left(1-e^{-\beta \Delta {\mathrm{ozone}}_{s}}\right)$$ 3

where *y*_*c*,*s*_ is the estimated change in annual respiratory-related deaths per grid cell *c* and scenario *s*, β is the risk coefficient associated with O_3_ exposure, and Δozone_*s*_ is the change in gridded ozone season MDA8O_3_ mixing ratio between each future scenario and the corresponding 2005–2014 reference scenario. Incidence_*c*_ and population_*c*_ refer to gridded estimates of the baseline respiratory mortality rates and total population counts for ages 0–99, respectively, averaged over the 2090s decade (2085–2094).

For the change in the PM_2.5_ health burden, BenMAP uses a nonlinear function based on the absolute PM_2.5_ concentrations in the reference and future scenarios (eq 4)­

$$y_{c,s}={\mathrm{incidence}}_{c}\cdot {\mathrm{population}}_{c}\cdot \left(1-e^{-\beta \cdot \left(T\left(Q_{b}\right)-T\left(Q_{s}\right)\right)}\right)$$ 4

where *y*_*c*,*s*_ is the estimated change in annual NCD + LRI-related deaths per grid cell *c* and scenario *s*. β is a parameter calculated when approximating the Fusion model RR function, and *Q*_b_ and *Q*_*s*_ refer to the PM_2.5_ concentrations in the baseline and future scenario gridded air quality surfaces, respectively. *T*(*Q*) refers to the arctangent approximation of the Fusion model RR, as outlined in eqs 1 and 2. Incidence_*c*_ and population_*c*_ refer to gridded annual estimates of the baseline background NCD + LRI mortality rates and total population counts for ages 25+, averaged over the 2090s decade (2085–2094). As previous studies have found that population aging will likely increase the future health burdens from air quality,^9^ we focus this analysis on the population age structure predicted for the 2090s decade to account for the age distribution in each country by the end of the century.

### Future Changes by Degree of Warming and Physical and Monetized Impacts

Lastly, by isolating the impacts of meteorology in the GEOS-Chem simulations, we can use the resulting mortality estimates to develop impact by degree functions in a reduced form tool for each GCM, country, and pollutant (4800 functions total). In this work, we create functions by relating the change in country-level O_3_- and PM_2.5_-related annual deaths per capita to the global average temperature change in each future scenario relative to the reference period (2005–2014), while holding population counts at 2090 levels. By indexing dynamic and complex mortality changes to global temperature in this way, impact by degree functions help to translate scenario-based impact results into time-independent generalized functions that can be applied to any future scenario.^47^ This generalization helps facilitate comparisons across studies and improves understanding and communication of results.^48^ In this work, we run the reduced form tool 10 000 times, using probabilistic inputs of temperature (see Supporting Information Text S3), population, and GDP from the RFF-SP scenarios^44^ to generate time series (2020–2300) of changes in future annual mortality estimates in each country. The RFF-SP scenarios generally encompass the range of total population and GDP values estimated in the various SSP scenarios.^44^ As the reduced form functions are developed from the per capita mortality estimates from BenMAP (described above), the reduced form tool implicitly assumes the same age distribution of the total population (i.e., 2090s decade). Annual mortality cases in each country are then monetized to estimate annual impacts at the end of the century in units of 2024 U.S. dollars by using the income-adjusted Value of a Statistical Life (VSL) (Supporting Information Text S3). Annual monetized values from 2020 to 2300 are then used to calculate the net present value (NPV) of the change in air quality mortality associated with the temperature change from an additional ton of CO_2_ emissions (using a 2% Ramsey discount rate), and compared to previous studies.^39,49,50^ Additional details about the development and assumptions underlying the impact by degree function, the monetization approach, NPV calculations, and the RFF-SP scenarios are provided in Supporting Information Text S3.

## Results and Discussion

### Meteorology-Driven Changes in O_3_ and PM_2.5_ Concentrations

Relative to the 2005–2014 period, the SSP2-4.5 GCM–CTM results in Figure 2 reveal that changes in MDA8O_3_ and annual PM_2.5_ concentrations are projected to vary widely across regions. While there are small global changes on average (Figure 2), ΔMDA8O_3_ over land ranges from −4 to +12 ppbv in the CESM-driven CTM simulation and −5 to +6 ppbv in the GISS-driven simulation (Table 1), which is similar to but slightly smaller than the range (−8 to +10 ppbv) previously reported across the U.S., Asia, and Europe.^51^ For PM_2.5_, changes in global land average concentrations relative to the reference scenario are also close to 0 μg/m^3^, but range from −15 to +3 μg/m^3^ in both GISS and CESM-driven CTM simulations (Table 1), illustrating significant sensitivities of regional PM_2.5_ concentrations to future changes in meteorology. Potential factors driving these predicted changes are discussed next.

**Figure 2:**
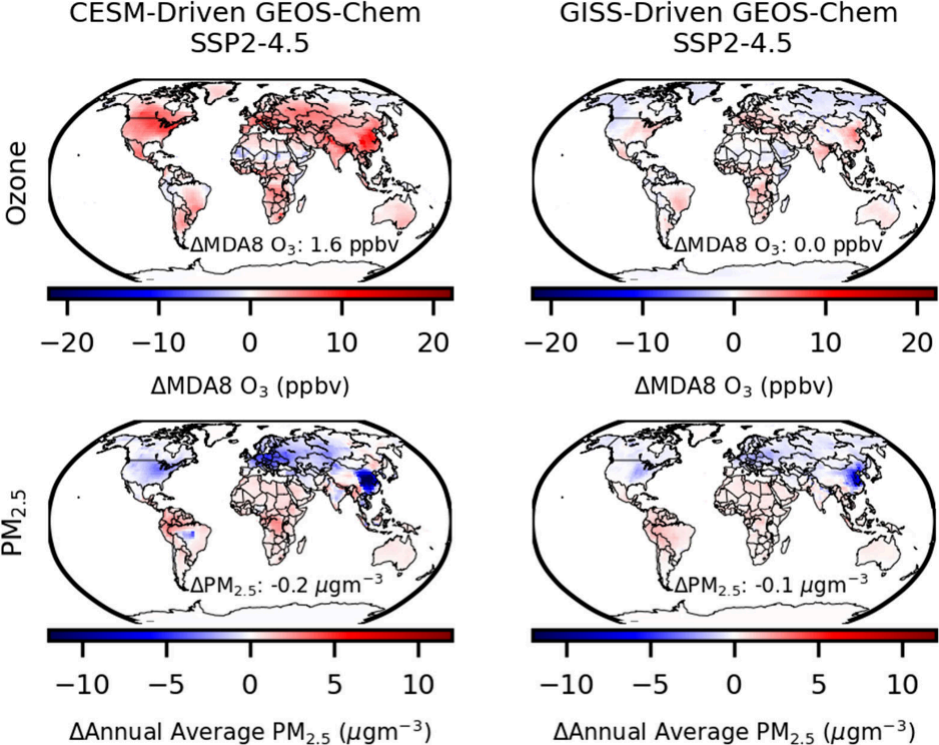
Change in MDA8O_3_ (top row) and PM_2.5_ (bottom row) concentrations in the 2090s decade in the SSP2-4.5 CESM- (left) and GISS-driven (right) CTM simulations, relative to the 2005–2014 period. Global average concentration changes (land average) are in the bottom left of each panel. Figures S3 and S4 provide results for all assessed SSP scenarios.

### Ozone: Global and Regional Concentration Changes

Consistent with previous studies,^8,10,25,52,53^ global ozone season ΔMDA8O_3_ is generally estimated to increase in the future with increasing temperatures (Figure S3), however, future ΔMDA8O_3_ is also estimated to decrease throughout parts of Northeastern Russia, Alaska, western Canada, and parts of the Middle East, Africa, and South America. There are also differences in the magnitude of estimated ΔMDA8O_3_ between the CTM simulations driven with meteorological parameters derived from the CESM vs GISS models (Figure 2, top row). In general, the direction (positive vs negative) of estimated ΔMDA8O_3_ is similar between the GISS and CESM-driven simulations, but the magnitude of ΔMDA8O_3_ in the CESM-driven simulations is larger and more positive than in the corresponding GISS-driven simulations. This is partially due to the higher temperatures estimated to occur in the CESM-driven simulations in each respective SSP scenario (Table S1) due to a larger equilibrium climate sensitivity in the CESM2 model relative to GISS.^54^

To better understand these results, we compare simulated ΔMDA8O_3_ to the GEOS-Chem diagnostic outputs for a series of relevant meteorologically driven processes. As presented in Table S3 and discussed elsewhere,^8,14,51,53^ multiple processes in the troposphere have nonlinear impacts on O_3_ production, transport, and loss, which can lead to either net increases or decreases in future O_3_ concentrations. While the influence of any one factor on ΔMDA8O_3_ cannot be reduced to a single global trend (global correlation coefficients are <0.1), by investigating the spatial correlations between changes in ΔMDA8O_3_ (Table S3) and each process (Figures S5 and S6), we can better understand the sensitivities of net ΔMDA8O_3_ to meteorological changes, both globally and regionally.

Figures S5 and S6 show that globally, the estimated increases in ΔMDA8O_3_ generally correspond to the increase in estimated global surface temperatures and tropospheric stability (taken as the change in the potential temperature difference between the top and bottom of the troposphere^52^). This result is consistent with previous studies that have also often found the strongest correlations between temperature and ΔMDA8O_3_,^8^ particularly in polluted regions.^12,14^ However, simulated decreases in future ΔMDA8O_3_ spatially correspond to multiple factors that decrease tropospheric O_3_ concentrations, such as increases in atmospheric water vapor that lead to net O_3_ destruction (particularly in less polluted, low NO_*x*_ regions), increases in planetary boundary layer (PBL) height that can increase dilution through mixing and ventilation, increases in dry deposition velocity that increase O_3_ loss to the surface, as well as decreases in tropospheric stability that can also increase mixing. Specifically, decreases in ΔMDA8O_3_ in Africa and parts of South America correspond to increases in horizontal winds, which may indicate an increase in dilution in these regions. In the SSP1-2.6 driven simulation (Figures S5 and S6), the global average ΔMDA8O_3_ decrease may be the result of relatively small temperature-driven increases, offset by a higher sensitivity to increases in water vapor in less polluted regions.^12,13^ As the production of O_3_ requires the emissions of NO_*x*_ (particularly in rural regions with more limited anthropogenic sources), the large increases in modeled NO_*x*_ from lightning and soil in Figures S4 and S5 also generally correspond to the modeled regional O_3_ increases. However, in NO_x_-saturated regions, NO_x_ increases from these sources could lead to O_3_ reductions, such as in urban centers or remote environments. Sources of natural emissions such as BVOCs, lightning, and soils are highly uncertain, but continue to be increasingly important for understanding future O_3_ production regimes.^55,56^

From a regional perspective, previous studies have focused on parts of Europe, Asia, and the contiguous U.S. In Europe, models have consistently estimated that future meteorology will increase ozone season O_3_ concentrations in central and southern Europe and decrease O_3_ in northern Europe,^12,57,58^ due to estimated increases in temperatures and BVOCs (O_3_ increases), as well as increases in water vapor (O_3_ decreases) in less populated northern regions. Increases in O_3_ may also result from decreases in O_3_ deposition velocity due to future drought conditions in Europe.^59^ Overall, both the CESM- and GISS-driven simulations in this work agree with the geographic trends of previous studies and show the large ΔMDA8O_3_ increases over central and southern Europe and more moderate increases over northern Europe, such as Denmark and Sweden (Figure 2).

In Asia, previous modeling studies have predicted varying impacts on O_3_ throughout Asia, estimating both increases^13,25^ and decreases^60^ in future high O_3_ events. Previously simulated O_3_ increases in polluted regions and O_3_ decreases in parts of western China and southern India have been thought to be influenced by changes in the summertime monsoon,^12^ increases in clouds and precipitation,^60^ reduced background O_3_ from increased water vapor,^15,61^ and changes in the O_3_ deposition velocity from changes in soil moisture.^16^ The CESM- and GISS-driven CTM simulations (Figure 2) suggest that future changes in meteorology-driven processes will increase ΔMDA8O_3_ in this region, corresponding to more stagnant conditions and decreases in dry deposition velocity (Figure S6).

In the U.S., many modeling studies agree that future changes in meteorology will increase O_3_ over the Northeastern U.S., but agree less for the Midwest, and have little agreement as to whether O_3_ will increase or decrease in the Southeast and Westen U.S.^8,11,12,14,53^ Decreases in the Southeast are estimated to result from future increases in BVOCs, such as isoprene, leading to decreased O_3_ production efficiency and net O_3_ destruction in this region where O_3_ production may already be VOC saturated (or NO_*x*_ limited).^10,11,52,53^ Additional studies have also suggested that this negative correlation in the Southeast may results from changes in transport patterns (e.g., possible shift of Bermuda High) that reduce O_3_ through ventilation.^12,62^ In this work, increases in total organic aerosol mass in both GISS and CESM-driven simulations (Figures S5 and S6) suggest that the decrease in Southeast O_3_ is more likely driven by increases in BVOC emissions and water vapor rather than increased ventilation. In general, the results in this work suggest that the largest changes in ΔMDA8O_3_ are estimated to occur in the Northeast and Midwest U.S., with more moderate (or negative) increases in the Western and Southeastern U.S., depending on the SSP scenario and GCM.

### PM_2.5_: Global and Regional Concentration Changes

As shown in Figure 2 and Figure S3, annual average PM_2.5_ is estimated to decrease in each future SSP scenario, despite no change in the simulated emissions of anthropogenic air quality precursors. Like ΔMDA8O_3_, net changes in PM_2.5_ concentrations are dependent on multiple meteorologically driven processes that impact its production, transport, and loss (Table S4). These processes, however, are complicated by competing effects that lead to different trends across different PM_2.5_ components. For example, higher temperatures can increase BVOC emissions^63^ and SO_2_ oxidation rates,^64^ that lead to increases in organic carbon and inorganic sulfate aerosol mass, but that also decrease inorganic nitrate and ammonium aerosol mass due to increased thermodynamic partitioning to the gas phase.^8^ The net effect on total PM_2.5_ mass will therefore depend on the local composition of PM_2.5_ aerosol (i.e., the fraction of inorganic vs organic components) and how that composition is estimated to change in the future. In addition, since background concentrations of PM_2.5_ are low compared to O_3_, changes in PM_2.5_ are more sensitive to loss through wet deposition (primary loss mechanism) and changes in ventilation (stagnation, mixing height).^65–67^ Figures S7 and S8 show the change in annual average total PM_2.5_ mass and relevant influencing processes described in Table S4. Figure S9 (GISS) and Figure S10 (CESM) additionally show the percentage change of each PM_2.5_ component in the future simulations relative to the reference scenario.

Collectively, Figures S9 and S10 show that the inorganic and organic components of PM_2.5_ are estimated to have opposite trends in the future. Despite increases in natural sources of precursor emissions (Figures S5 and S6), worldwide decreases in inorganic components (nitrate, sulfate, and ammonium) are primarily due to rising temperatures that thermodynamically favor gas-phase species and estimated increases in loss through wet deposition. Some exceptions to these trends are in parts of Africa, South America, and Australia, which show moderate increases in inorganic compounds, potentially due to decreases in annual precipitation rates and increases in water vapor leading to sulfate increases (Table S4). In contrast, total organic aerosol is estimated to increase globally (Figures S9 and S10), particularly in the southern hemisphere where there are larger sources of BVOCs.

Since the health impact calculations only consider total PM_2.5_ mass, the net future effect of meteorology on total PM_2.5_ health impacts will be determined by how the changes in each component of PM_2.5_ (and their precursors) impact the total PM_2.5_ mass in each region. For example, the inorganic fraction of aerosol mass in highly populated locations is often greater due to local sources of nitrogen, sulfate, and ammonia that are emitted from transportation, industrial, and energy sources.^3^ In more rural or vegetated areas, PM_2.5_ often has a larger organic aerosol fraction. Figure S11 shows that in regions where the global population is exposed, the inorganic reductions are only minimally offset by increases in organic aerosol. We, however, do not consider changes in PM_2.5_ precursor emissions. Therefore, if controls for inorganic precursors are implemented in the future, then the potential for PM_2.5_ inorganic reductions at warmer temperatures will be reduced and the temperature-driven increases in organic aerosol may eventually offset the reductions and result in a net global increase of PM_2.5_ mass in the future.

Future changes in PM_2.5_ have been less well studied than O_3_. Due to the high sensitivity of PM_2.5_ to changes in future precipitation, transport, and the treatment of organic chemistry, previous studies have generally disagreed on the magnitude and sign of predicted changes. For example, in the U.S., previous studies have predicted general increases in PM_2.5_ across the U.S., but also decreases in some components.^8,13,66,68,69^ Figure 2 shows a decrease in total PM_2.5_ in the U.S., except for the Southeastern U.S. where decreases in inorganic components are offset by strong increases in organic aerosol. In Europe, previous studies have predicted net increases in sulfate and carbonaceous aerosol in central and southern Europe,^8^ whereas Figures S9 and S10 show decreases in all aerosol components. In China, recent studies have predicted increases in PM_2.5_ over eastern China,^13^ corresponding to reductions in PBL height and windspeed (i.e., stagnant conditions),^15^ as well as decreases in regions with estimated precipitation increases.^70^ In contrast, this work does not predict strong increases in stagnation in this region and estimates increases in wet and dry deposition, which both result in reductions in total PM_2.5_. On the global scale, two recent analyses of free running CMIP6-derived PM_2.5_ concentrations found global PM_2.5_ increases, due to large increases in organic aerosol from BVOC emissions, as well as dust and sea salt (but these studies excluded nitrate aerosol).^18,65^

Disagreement with previous studies likely results from a combination of modeled differences in meteorological patterns, our use of fixed present-day anthropogenic precursor emissions (which impact the PM_2.5_ composition and how total mass will change in the future), the CTM treatment of BVOC emissions, soil NOx, and isoprene oxidation chemistry (which leads to SOA production), or the components of PM_2.5_ considered in each study. For example, recent results from most free-running GCM’s do not include aerosol nitrate,^19,65^ but do include dust and sea salt, which both have large interannual variability.^57^ Given that global PM_2.5_ decreases in this study are driven by reductions in ammonium and nitrate aerosol (Figure S11), results are likely sensitive to modeled differences in nitrate thermodynamic partitioning^71^ and biogenic SOA formation. Previous studies have similarly found larger uncertainties in future PM_2.5_ concentrations from differences between atmospheric chemistry models and ensemble members than from meteorological variability.^72^ Therefore, model parameterizations of future chemical and physical processes will continue to have large impacts on the estimated impact of future meteorological changes on total PM_2.5_ mass.

### Changes in Air-Pollution-Related Mortality and the Monetized Impacts

Just as future changes in O_3_ and PM_2.5_ concentrations are highly sensitive to regional meteorology, the resulting exposure levels and attributable number of deaths also have strong regional variability (Figures 3 and 4). While O_3_-attributable respiratory-related deaths are estimated to increase in most countries, the number of deaths attributable to PM_2.5_ exposure may increase or decrease in the future, depending on the region. To further illustrate this point, panels surrounding Figures 3 and 4 show how respiratory-related (or all-cause) mortality is estimated to change in eight example countries, given simulated changes in future meteorological conditions and constant population. We present these results using the impacts by degree approach^22,47,48^ as a way to index the changes in mortality in each country (which are determined by the full suite of modeled meteorological changes), to the level of global warming that those changes occurred at. This approach allows us to capture important regional and nonlinear changes in meteorology, while also allowing for a more direct comparison of impact results across models, and for a larger number of possible future scenarios.

**Figure 3:**
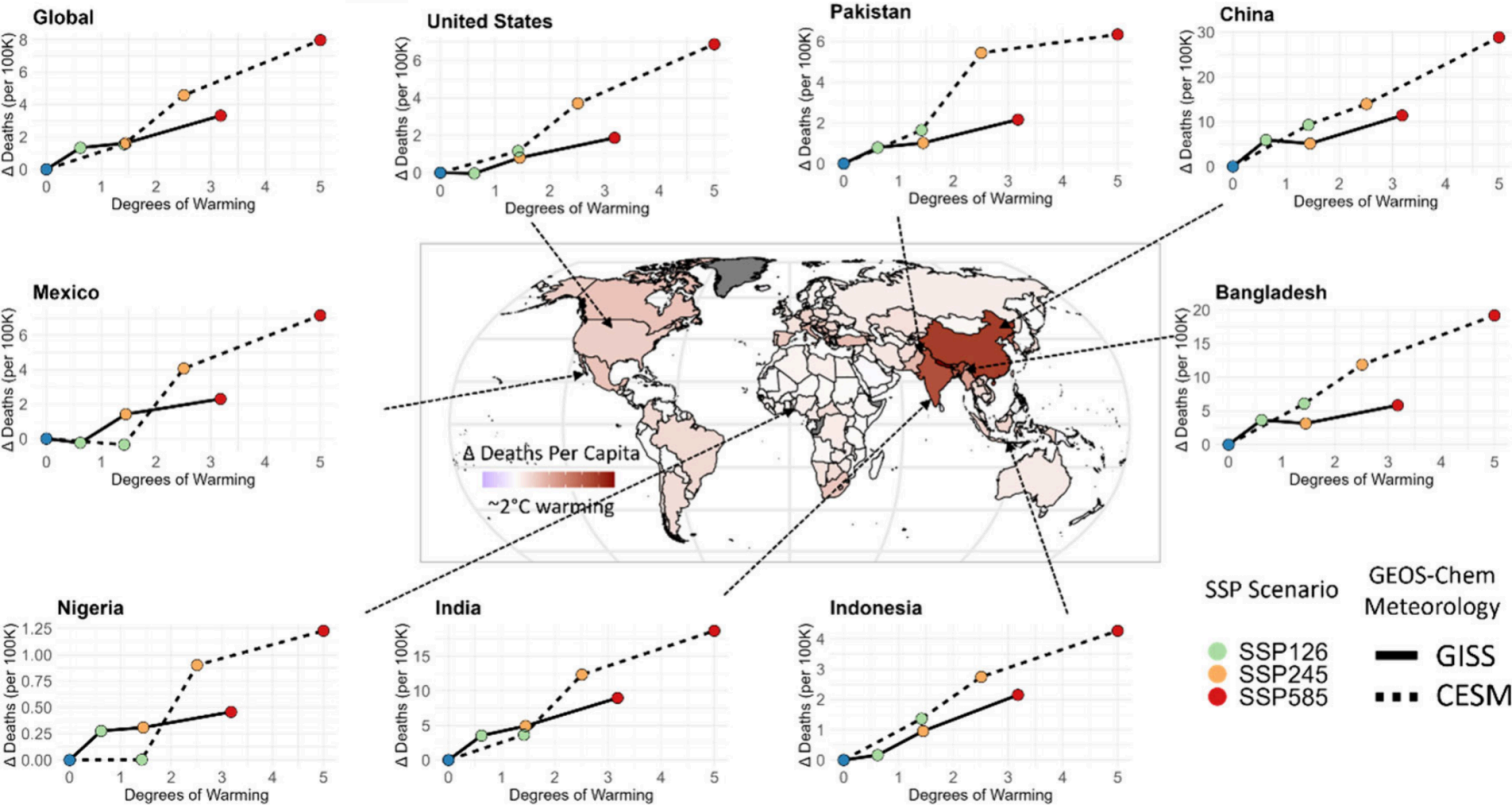
Changes in respiratory-related deaths (per 100 000) from meteorologically driven changes in MDA8O_3_, per degree of future global warming relative to 2005–2014. Map: change in deaths per capita in the SSP2-4.5 scenario (average GISS-driven and CESM-driven CTM results, corresponding to ∼2 °C of global temperature change). Surround plots: changes in respiratory-related O_3_-attributable deaths per degree of global temperature change for the 8 countries with the largest increases in the SSP2-4.5 scenario (calculated using constant 2090s population). Dashed and solid lines: results for the CESM- and GISS-driven CTM simulations, respectively. Point colors correspond to the SSP meteorological conditions used in each simulation.

**Figure 4:**
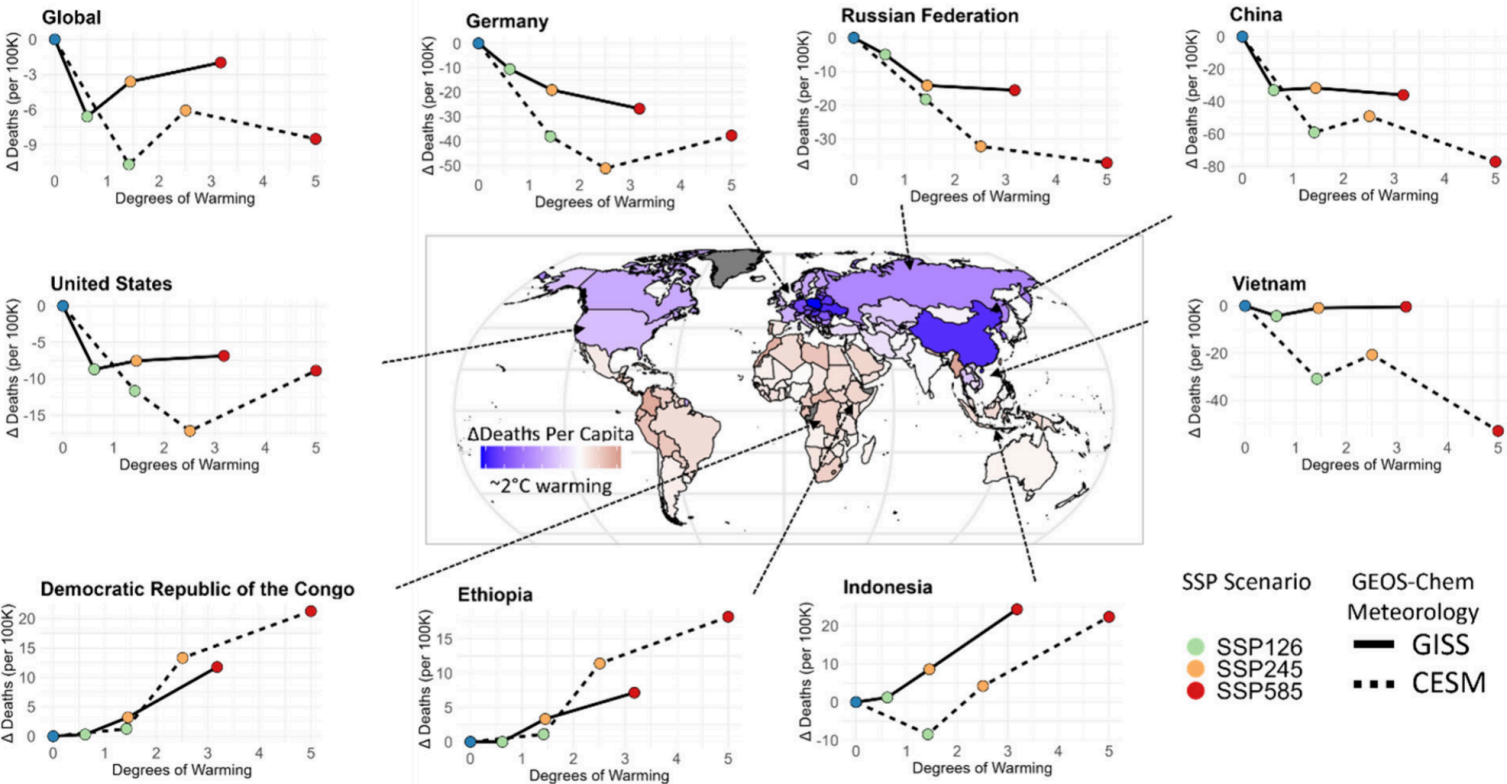
Changes in all-cause-related deaths (per 100 000) from meteorologically driven changes in PM_2.5_, per degree of future global warming relative to 2005–2014. Same as Figure 3 but for PM_2.5_. Surround plots show the per degree impacts for the 5 countries with the largest total decreases in PM_2.5_ attributable deaths, and the 3 countries with the largest increases.

In addition to regional variability, Figures 3 and 4 also show that future changes are estimated to have the opposite effect on PM_2.5_- and O_3_-attributable mortality rates. For example, changes in PM_2.5_ all-cause mortality rates are estimated to decrease with increasing temperatures in many countries in the Northern Hemisphere, whereas the same estimated meteorological changes are estimated to increase O_3_-attributable mortality in these same countries (i.e., United States, China, and India). For both pollutants, the sign and trends are generally consistent between the two GCMs, though there are often differences in magnitude at each given degree of warming. While the by degree framework is intended to minimize these differences and allow for more consistent comparison of impact results across models, future changes in PM_2.5_ and O_3_ are also highly sensitive to the precursor emissions in each scenario,^17,19,22^ which likely adds additional variability and uncertainty to the functions.

Lastly, we use the by degree impact functions for each model, pollutant, and country in a reduced form tool to estimate the physical and monetized impacts at the end of the century across 10 000 RFF-SP future scenarios.^44,73^ By averaging results across the 10 000 scenarios, we find that meteorologically driven changes in global air pollutant concentrations will decrease the number of annual deaths by 180 000 (95% CI: −460 000 to +180 000) in the year 2100 relative to the recent past (2005–2014). This reduction corresponds to an annual global benefit in 2100 of $7.3 trillion U.S. dollars (USD) (95% CI: $49T benefits to $16T damages). The 95% confidence interval represents the 2.5th and 97.5th percentiles of the net impacts across the 10 000 RFF-SP scenarios, averaged across the two GCMs. These results account for future changes in meteorology and total national-level population, and reflect the age distribution of the national population in each country in the 2090s decade. The latter is an important consideration as previous studies have shown that future mortality burdens are sensitive to population aging.^9,74,75^

As global average concentrations of O_3_ and PM_2.5_ are estimated to change in opposite directions, annual O_3_-related deaths are similarly estimated to increase by 230 000 in 2100 (95% CI: 70 000 to 480 000), valued as an annual loss of $3.8T USD (95% CI: $3.2T to $17.7T), while annual PM_2.5_-related deaths are estimated to decrease by 410 000 in 2100 (95% CI: 200 000 to 610 000), valued as a benefit of $11.2T USD (95% CI: $1.4T to $49.7T). Not all countries share these global trends. For example, in the U.S. and in China, the annual deaths are estimated to decrease by 20 000 and 240 000 by 2100, respectively, but the annual deaths in India are estimated to increase by 40 000 by 2100. This results from the increase in O_3_-related mortality in India offsetting the more moderate reductions in PM_2.5_-related mortality. Of the 201 countries included in this study, only 60% are estimated to experience net decreases in annual air quality-related mortality under future meteorological conditions relative to the recent past (2014–2005), which highlights the importance of considering regional changes for better understanding future impacts.

A very limited number of previous studies have isolated (nonfire) meteorological impacts on future changes in PM_2.5_- and O_3_-related mortality. A previous comparison of multiple free-running CMIP5 GCMs found a large spread across models, with both increases and decreases of both pollutants, but an average increase in both global PM_2.5_ (215 000) and O_3_ (44 000) attributable deaths annually by 2100.^24^ The study authors attributed the GCM spread to differences in large-scale meteorology, and in the model treatment of atmospheric chemistry and feedback processes. A similar study of downscaled and bias corrected O_3_ concentrations from three free-running CMIP6 GCMs also found that climate change alone (including wildfire effects) increased global O_3_-related mortality in 2090 in the single SSP3-7.0 scenario by up to 217 000.^25^ Two global studies using results from the GFDL AM3 chemistry-climate model and a previous version of the GEOS-Chem CTM also estimated increases in O_3_ mortality in many populated regions (though a net global decrease),^28^ and global increases in both O_3_ and PM_2.5_ mortality^27^ from meteorological changes in the IPCC SRES A1B scenario. In China, a previous study^15^ using a different GCM and CTM also found increases in both meteorologically driven PM_2.5_- (including dust) (27 000) and O_3_-related (35 000) deaths annually, largely attributed to increases in atmospheric stagnation events and wintertime haze. In the U.S., a previous study^22^ using meteorological conditions from two CMIP5 GCMs and a high resolution CTM similarly found net increases in both meteorologically driven O_3_- (2100) and PM_2.5_ -related (not including dust) (210 000) deaths annually by 2100. Differences in the GCMs, meteorology, CTMs, exposure CRFs, and population projections in each study could explain some of the differences in magnitude in the estimated changes in O_3_-related mortality. However, the decreasing trend we find in PM_2.5_-related mortality, both globally and in populated regions such as the U.S. and China, is not consistent with the majority of previous studies. Differences with global studies^24^ may be due to the inclusion of different aerosol components (e.g., inclusion of nitrate, exclusion of dust) or treatment of atmospheric chemistry in a CTM comparted to GCMs. Differences in the modeling approach and assumptions (discussed in the next section) may be driving the differences in PM_2.5_ sign in the U.S. or China.

As described in Supporting Information Text S3, we discount and integrate the annual stream of monetized deaths between 2030 and 2300 from the output of the reduced form tool to derive the NPV per ton of CO_2_ emissions change associated with meteorologically driven changes in PM_2.5_- and O_3_. This follows the same approach as recent calculations of the NPV of future environmental impacts.^39,50,73^ Globally, the NPV derived using a 2% Ramsey discount rate is −$15 per metric ton of CO_2_ emitted (median, −$2/mt of CO_2_; 90% CI, −$103 to +$30/mt of CO_2_). This represents the average NPV across the two GCMs and the 10 000 probabilistic future RFF-SP scenarios. The median and confidence interval represent the 50th, 5th, and 95th percentiles across the probabilistic scenarios. For O_3_, the average global NPV is $23/mt of CO_2_ (median, $20/mt of CO_2_; 90% CI, +$7 to +$45/mt of CO_2_) and −$38/mt of CO_2_ (median, −$24/mt of CO_2_; 90% CI, −$118 to −$3/mt of CO_2_) for PM_2.5_. The sign of the O_3_ NPV is consistent with a previous study that found a net economic global welfare loss (defined as macroeconomic consumption and the value of leisure time) from meteorologically driven changes in future O_3._ ^28^ The NPV values for both pollutants in this work are smaller than the latest global NPV estimate of $260/mt of CO_2_ (in 2024 dollars), which represents the cost associated with a broader range of economic damages from environmental, health, agricultural, and infrastructure impacts from a 2030 pulse of CO_2_ emissions.^49,76^ This comparison suggests that incorporating the benefits from this study would lead to a 6% reduction in the latest global NPV estimates. Consistent with the concentration and mortality results, the NPV also has striking geographic variability (Figure S12), where the PM_2.5_-related benefits offsetting the O_3_-related damages in all regions except for Africa, the Middle East, South Asia, and Latin America and the Caribbean. In the U.S, the total NPV is −$3/mt of CO_2_ (median, −$2/mt of CO_2_; 90% CI, −$15 to +$5/mt of CO_2_), ranging from $3/mt of CO_2_ for O_3_ to −$6/mt of CO_2_ for PM_2.5_. This suggests that the U.S. NPV may be roughly 25% lower than the most recent domestic estimates.^50,77^

### Sensitivities and Uncertainties

Given sensitivities in the number of models and underlying assumptions used, it can be challenging to compare results across studies. For example, differences in the sign of the ΔPM_2.5_ could be explained by differences in the version of the GCMs (CMIP5 vs CMIP6), the horizontal resolution, parameterizations in the CTM compared to a GCM, and projections in temperature change and population between studies. As this study only provides the second assessment of the isolated meteorological-driven air quality impacts from a coupled CMIP6 GCM–CTM,^23^ future work should assess the sensitivity of the sign of the PM_2.5_ result to the most recent GCM meteorological trends, the meteorology downscaling approach, representation of interactive fires and mineral dust, and the CTM selection and resolution.

Alternatively, we can assess the sensitivity of the mortality results in this work to select uncertain parameters. For example, while we cannot assess the sensitivity or uncertainty associated with the CTM or GCM era (i.e., CMIP5 vs CMIP6), we can compare results across the meteorological inputs from two different GCMs, as well as the 95th percent confidence interval associated with the CRF’s used in BenMAP, and uncertainties in future projections of temperature and population. Table S5 shows that all sensitivity tests consistently predict global decreases in PM_2.5_-attributable mortality and global increases in O_3_-attributable mortality by 2100. Both O_3_- and PM_2.5_-attributable mortality results are moderately sensitive to assumptions in the CRFs, while the PM_2.5_ results are more highly sensitive to the GCM selection. This reflects the general agreement that future meteorology will increase O_3_ concentrations, and more limited agreement across PM_2.5_ studies. Table S5 also shows that both PM_2.5_- and O_3_-attributable mortality is most sensitive to assumptions in future temperature. Table S5 shows that when considering the 90th percentile of future possible temperatures, the increases in PM_2.5_- and O_3_-attributable mortality are such that the total change is predicted to be a net increase in air-pollution-related mortality by 2100 rather than a net decrease. Not shown in Table S5 is the additional uncertainty introduced by the minimum exposure threshold for O_3_, which changes the global impact by <1% in most cases but could introduce larger uncertainties in select coastal countries with the lowest O_3_ concentrations (Text S2). While these results do not test all aspects of uncertainty, they do collectively suggest that the robustness of this result could be improved by prioritizing the incorporation of meteorology from additional GCMs or ensemble members, different CTMs, or narrowing the uncertainty range of future temperature scenarios.

Additional limitations in this analysis include the assumption of constant concentrations of dust and sea salt between the baseline and future scenarios, as well as a lack of changes in precursor emissions. Future changes in dust and sea salt are highly variable, depending on modeled parameterization of natural feedbacks, and may increase in the future.^18,23^ Similarly, changes in wildfires are highly uncertain and are intentionally excluded here, but are estimated to contribute to net increases in future O_3_- and PM_2.5_-attributable mortality.^78–80^ These impacts from fires are entirely independent from the other meteorological changes assessed here, and could be added to the impacts described above. This study also did not consider how changes in baseline precursor emissions will impact the meteorological sensitivity of PM_2.5_ and O_3_ concentrations.^11,22^ As the PM_2.5_ decreases in this study are largely being driven by reductions in inorganic aerosol (which primarily have anthropogenic sources) (Figure S11), it follows that future pollution mitigation measures aimed at reducing PM_2.5_ precursors would also reduce the absolute benefits that are estimated to result from PM_2.5_ mass reductions in warmer futures. As natural emissions and organic aerosol mass continue to increase with warmer temperatures (Figure S11), it follows that there could be a crossover point where increases in organic mass could outweigh the inorganic mass decreases and result in a net increase in PM_2.5_ attributable mortality in the future.

We also do not explicitly account for future changes in background O_3_ concentrations associated with changes in methane emissions.^39^ However, any changes in background O_3_ associated with atmospheric water vapor, lightning NO_*x*_, and entrainment of stratospheric air, particularly in less polluted regions, will be captured. In addition, we only consider changes in surface concentrations, not the total atmospheric column,^65^ and the coarse (2° × 2.5°) horizontal resolution of the CTM may not be sufficient to resolve all atmospheric transport or precipitation patterns in areas with complex terrain or coastlines, and may not capture chemical changes at the urban scale,^81^ though this latter effect is reduced by implementing a bias correction (Supporting Information Text S1). Therefore, this analysis is best suited to consider changes in long-term average exposure and may not fully resolve short-term pollution events at the urban scale.

Despite the computational complexity and various sources of uncertainty, this is the first study to use the meteorological output from two CMIP6 GCMs with a CTM to isolate the impact of future changes in meteorology and population on future mortality and monetized impacts resulting from changes in exposure to PM_2.5_ and O_3_. Consistent with previous studies, the future changes in meteorology and natural feedbacks are estimated to increase the mortality associated with O_3_. In contrast to previous studies with CMIP5 GCMs, simulations in this work suggest that these same changes in future meteorology will result in decreases in PM_2.5_ mortality, which outweigh the O_3_ increases and result in 180 000 fewer deaths in 2100 relative to the recent past, an annual net benefit of ∼$7T USD. Detailed assessments show the sensitivity of these changes to regional changes in meteorological parameters and individual PM_2.5_ components and reveal a high level of variability between countries, which highlight the need to consider regional and local influences of meteorology and natural feedbacks when estimating how air pollution impacts may change in the future.

## Supplementary Material



## Data Availability

For GCAP and GEOS-Chem code availability, see ref 23. GISS and CESM meteorological data are available for download at http://atmos.earth.rochester.edu/input/gc/ExtData/GCAP2/CMIP6/. BenMAP code is available on Zenodo: 10.5281/zenodo.17419632. The code for the reduced form model is available on GitHub: https://github.com/USEPA/globalAQ_rft.

## Notes

Additional methodological details, including on the global modeling of PM_2.5_ and O_3_ concentrations, mortality estimates, monetization approach, and impact by degree functions, and detailed results, including detailed trends in PM_2.5_ and O_3_ concentration changes with meteorologically driven variables and results from sensitivity calculations (PDF)
