# Supplementary material for: Meteorologically Driven Changes in Future Global Air Quality: Physical and Monetized Impacts
Source: Environ Sci Technol. 2026 Jul 2;60(27):19051–63. doi: 10.1021/acs.est.5c14713 (PMC13374105; doi:10.1021/acs.est.5c14713)
Supplement: Supplementary file 1 [file es5c14713_si_001.pdf]

Supporting Information for:

Meteorologically Driven Changes in Future Global Air Quality: Physical and Monetized Impacts

Erin E. McDuffie[1], Lee T. Murray[2], Sebastian D. Eastham[3], Melanie Jackson[4], Marcus C. Sarofim[1], William Raich[4], Richard Burnett[5], Jim Anderton[4], Henry Roman[4], James E. Neumann[4], Simone Tilmes[6], Kwesi A. Quagraine[6], Neal Fann[7]*

[1]EPA Office of Atmospheric Protection, Washington, DC, 20460, USA
[2] Department of Earth and Environmental Sciences, University of Rochester, Rochester, NY, 14627, USA
[3] Department of Aeronautics, Imperial College London, London, SW7 2AZ, United Kingdom
[4] Industrial Economics, Inc., Cambridge MA, 02140, USA
[5] 164 Fanshaw Avenue, Ottawa, ON, K1H6C9, Canada,
[6] NSF National Center for Atmospheric Research, Boulder, CO, 80305, USA
[7] Harvard University, Cambridge MA, 02138, USA
* Corresponding author

Contents in this File:

Text S1-S3

Figures S1-S10

Tables S1-S4

Equations S1 -11

Introduction

This supporting information provides the supplemental figures, tables, and text sections that are referenced in the main text, including detailed information on the global modeling of atmospheric $PM_{2.5}$ and O_3 concentrations, BenMAP mortality estimates, monetization approach, and reduced-form tool.

Supporting Information Text S1. Global Atmospheric Modeling Details

Each General Circulation Model (GCM) is driven by the emissions from a single SSP scenario. As described extensively elsewhere, the SSPs or Shared Socioeconomic Pathways were developed to describe a set of possible future scenarios for global society over the next century, ranging from SSP1, a world that focuses on sustainability, mitigation, and adaptation, to SSP5, a fossil-fueled driven world that faces challenges to adapt and mitigate. SSP2 is often referred to as the middle of the road scenario that reflects the continuation of current development patterns. Within each socioeconomic world, there are multiple possible emission pathways that lead to different levels of radiative forcing, which are indicated by the second number. For example, 2.6 is generally considered to be a 2°C warming scenario, 4.5 is considered a middle level of warming, and 8.5 is a high warming scenario. For the same emission pathways, the actual modeled changes in temperature at the end of the century will vary by GCM, based on the underlying parameterizations of physical and chemical processes and feedbacks. For computational efficiency and to assess the widest range of possible future temperatures, we use the GCM output from three SSP scenarios representing a low, medium, and high amount of pollutant precursor emissions and warming by the end of the century, SSP1-2.6, SSP2-4.5, and SSP5-8.5. Notably, we do not assess the results from SSP3-7.0 as this scenario was developed specifically to assess the impact of reduced air pollution mitigation and has higher O_3 and aerosol precursor emissions than SSP5-8.5.

The set-up for the GEOS-Chem chemistry-transport model (CTM) is the same as that previously described in Murray et al., (2024)[1], but new simulations driven by the GISS and CESM meteorological inputs were run for this analysis. For each simulation, the GEOS-Chem CTM was initialized over a 15-year period at a 4°×5° resolution and then run for 10 years at the 2°×2.5° native model resolution. The transport and chemistry time steps in each simulation were set to 10 and 20 minutes respectively, to optimize simulation accuracy and computation efficiency. GEOS-Chem was also run using the 'fullchem' chemical mechanism, which is a unified coupled aerosol-oxidant chemistry in the troposphere and stratosphere, and a linearized mechanism in the mesosphere. We use the simple, irreversible, direct yield scheme for the secondary organic aerosol (SOA) component of $PM_{2.5}$ for improved global performance and computational efficiency[2]. Sulfate, nitrate, and ammonium components of $PM_{2.5}$ are calculated using the ISORROPIA v2.2 thermodynamic module, with coupled gas-phase and in-cloud sulfur oxidation.

To isolate the influence of future meteorological conditions, sources of anthropogenic and wildfire emissions of air pollutant precursors (CO, NO_x, NH_3, SO_2, BC, OC, NMVOCs) are set to average values from the recent past (2005-2014) in all GEOS-Chem simulations, as derived for the CMIP6 experiment[3]. For purposes of chemistry, methane is set as a surface boundary condition using the 2005-2014 average

values but is allowed to advect and react. However, the meteorology inputs from the GCMs include the radiate effects of future methane abundances in each scenario. In contrast, natural sources of emissions, including NO_x from lightning and soil microbial activity, as well as terrestrial and marine biogenic emissions of non-methane volatile organic compounds (NMVOCs), which are dependent on local meteorological conditions, are calculated by GEOS-Chem during runtime. Additional emissions of SO_2 from volcanoes and NH_3 from wild animals are assumed to be invariant with time.

GEOS-Chem outputs of hourly surface O_3 mixing ratios and monthly concentrations of $PM_{2.5}$ (excluding dust and sea salt) from each simulation were archived and regridded to 0.5°×0.5°, as described previously[1]. For O_3, we use the standard diagnostic outputs of hourly surface O_3 mixing ratio (ppb). For the health impact modeling, we use these gridded hourly outputs to calculate the average ozone season maximum daily 8-hour average O_3 ($MDA8O_3$) exposure for each 10-year simulation, by averaging the annual maximum of the 6-month running mean of the monthly $MDA8O_3$ for each 10-year time-period. To account for possible climatological bias in the GEOS-Chem simulations[4], results from each simulation (historical and future) are corrected to independent constraints of average 2005-2014 gridded ozone season $MDA8O_3$ reanalysis product from DeLang et al., (2021)[5]. This bias correction is assumed to be invariant over time.

For $PM_{2.5}$, we calculate the monthly mean $PM_{2.5}$ concentration (μg/m^3) using a custom GEOS-Chem diagnostic that reports the sum of the individual inorganic and organic components of $PM_{2.5}$, scaled by their recommended 35% RH hygroscopic growth factors, but excluding contributions from dust and sea salt. Due to the large uncertainties in future concentrations of dust and sea salt aerosol[4], we follow Murray et al.[1] and calculate the present-day gridded concentrations of dust and sea salt using the satellite-derived GWR v4GL03 $PM_{2.5}$ product[6]. We assume that concentrations of dust and sea salt aerosol are constant over time and add these concentrations to the simulated $PM_{2.5}$ mass from each scenario. Similar to O_3, $PM_{2.5}$ concentrations from each historical and future simulation are bias corrected to independent constraints of average 2005-2014 gridded annual $PM_{2.5}$ from the satellite-derived $PM_{2.5}$ product[6].

Table S1. GCM scenarios used to drive the GEOS-Chem CTM. Each scenario represents an average of 10 simulated years. The GCM ensemble members are listed as the scenario variant

Scenario Number	GCM Name	Scenario	Ensemble member (Variant)	Average Temperature Change (°C) [a]
1, 2	GISS, CESM2	Historical	r1i1p1f2	-
3	GISS	SSP1-2.6	r1i1p1f2	0.6
4	GISS	SSP2-4.5	r1i1p1f2	1.4
5	GISS	SSP5-8.5	r1i1p1f2	3.2
6	CESM	SSP1-2.6	r1i1p1f2	1.4
7	CESM	SSP2-4.5	r1i1p1f2	2.5
8	CESM	SSP5-8.5	r1i1p1f2	5.0

[a] Area-weighted changes in temperature for the global average relative to the recent historical past

Supporting Information Text S2. Adaptation of the Fusion risk model for BenMAP

The Fusion[7] risk model, $\Im(z)$ for concentration z, is a spline with two knots (μ, θ), composed of a linear term for concentrations $z < \mu$, a term whose derivative declines with concentration between μ and θ, and a term whose derivative declines by the inverse of concentration when $z > \theta$. Mathematically we represent this by

$$\frac{\partial \ln \Im(z)}{\partial z} \equiv \gamma D_{\Im}(z) = \gamma \begin{cases} 1 & z \leq \mu \\ \left(1 + \frac{1-\rho}{\rho}\left(\frac{z-\mu}{\theta-\mu}\right)^{\lambda}\right)^{-1} & \mu \leq z \leq \theta \\ \frac{\rho\theta}{z} & z \geq \theta \end{cases} \quad \text{Eq. S1}$$

where $\lambda = \frac{(\theta-\mu)}{\theta(1-\rho)}$, defined such that the second derivative is continuous at z= θ, with

$$\Im(z) = \exp\left(\gamma \int_0^z D_{\Im}(x)\partial x\right) =$$

$$\exp\left(\gamma\left(\min(z,\mu) + \int_{\mu}^{\min(\max(z,\mu),\theta)}\left(1 + \frac{1-\rho}{\rho}\left(\frac{x-\mu}{\theta-\mu}\right)^{\frac{\alpha(\theta-\mu)}{\theta(1-\rho)}}\right)^{-1} dx + \rho\theta \ln\left(\frac{\max(z,\theta)}{\theta}\right)\right)\right) \quad \text{Eq. S2}$$

The Fusion model function is continuous over all concentrations and exhibits different behavior beyond concentration thresholds μ and θ, generally representing the low and high end of exposure ranges observed in cohort studies. At low concentrations until concentration μ, the Fusion model function's derivative is equal to a positive constant γ. For the range of concentrations included in cohort studies, the function declines as concentration increases. Above concentration θ (representing concentrations outside of those observed in cohort studies), the derivative of the function declines as the inverse of the concentration.

To increase statistical stability, particularly for regions where accurate mortality data may not be available by age or disease, the Fusion model has estimated hazard ratios for all non-accidental cardiovascular and non-malignant respiratory causes of death for all ages 25-99. While this model does not apply a distinct effect coefficient by age, both the baseline health incidence and exposed population counts vary by age.

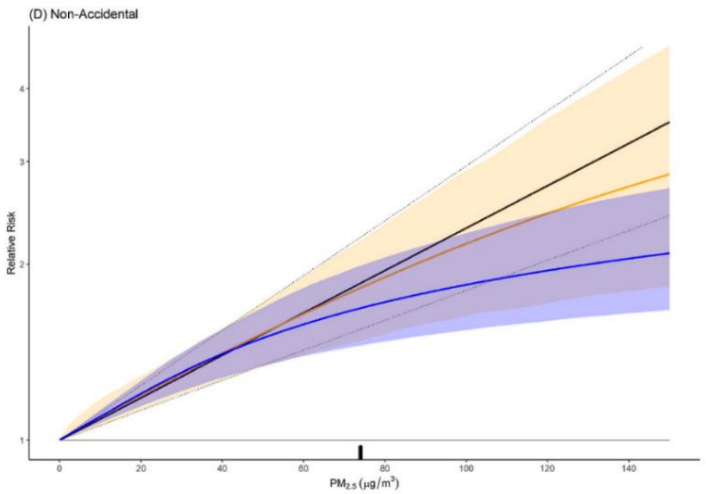

Figure S1. Comparison of Relative Risk predictions over $PM_{2.5}$ Concentrations for Non-Accidental Mortality. Fusion model in blue, GEMM in orange, and Log-Linear in black. Figure from Burnett et al.[7].

BenMAP relies on concentration-response functions for the relative risk based on the log-linear model: $\ln LL(z) = \gamma z$. Data inputs include the specification $\gamma \sim N\left(\hat{\gamma}, \hat{\sigma}_{\hat{\gamma}}\right)$. BenMAP can accommodate a single parameter to vary at random, such as γ. The Fusion model, for example, is characterized by three parameters (γ, μ, ρ) where uncertainty is quantified[7].

To use the Fusion model for benefits or burden analysis, one has to calculate $M = 1000$ benefits estimates, one for each of the $M = 1000$ curves generated by $M = 1000$ sets of parameter estimates. Therefore, BenMAP would have to be run 1000 times, a potentially impractical effort.

Since Fusion models the derivative of the logarithm of the relative risk, we seek to approximate $\frac{\partial \ln \mathcal{F}(z)}{\partial z} \equiv \gamma D_{\mathcal{F}}(z)$ with $\frac{\partial \ln \Im_A(z)}{\partial z} \equiv \gamma D_{\Im_A}(z)$, where $\mathcal{F}_A(z)$ denotes our approximate model, such that $D_{\Im_A}(0) = 1$, implying $\gamma D_{\Im_\alpha}(0) = \gamma D_{\Im_A}(0)$. That is, the derivative of our approximate model is the same as the derivative of the Fusion model at the origin. We then have

$$\ln \Im_A(z) = \gamma \int_0^z D_{\Im_A}(x) \partial x \equiv \gamma T_\omega^{\mathcal{F}_A}(z) \qquad \text{Eq. S3}$$

where $T_\omega^{\mathcal{F}_A}(z)$ is a monotonic transformation of z indexed by a vector of parameters ω. $\ln \Im_A(z)$ is characterized in a manner similar to the log-linear model, with a single randomly generated parameter, γ, multiplied by a transformation of concentration, in place of the concentration itself. BenMAP has capabilities to create such transformations of concentration.

We first find an appropriate functional form for $D_{\mathfrak{I}_A}$ to closely approximate the mean of the M model predictions, $\overline{D}_{\mathfrak{I}_A}$, over the observed concentration range of interest. Consider the form

$$D_{\mathfrak{I}_A}(z) = \sum_{j=1}^{J} \varphi_j \left(1 + \left(\frac{z}{\eta_j}\right)^2\right)^{-1} \qquad \text{Eq. S4}$$

where $\varphi_j = (\phi_j/\eta_j)/\sum_{j=1}^{J}(\phi_j/\eta_j)$, with $D_{\mathfrak{I}_A}$ a similar form to $D_{\mathcal{F}}(z)$ when $\mu < z < \theta$ and $\lambda = 2$. Here $D_{\mathfrak{I}_A}(0) = 1$ since $\sum_{j=1}^{J} \varphi_j = 1$. We selected this form since it has a definite integral

$$\int_0^z D_{\mathfrak{I}_A}(x)\partial x = \sum_{j=1}^{J} \tau_j \text{atan}\left(\frac{z}{\eta_j}\right) \equiv T_{\omega}^{\mathcal{F}_A}(z) \qquad \text{Eq. S5}$$

where $\tau_j = \phi_j/\sum_{j=1}^{J}(\phi_j/\eta_j)$. Estimates of $\omega = (\phi_l, \eta_l, l = 1, \ldots, L)$, are determined by non-linear regression.

We want to capture all the uncertainty in the Fusion model predictions among the M curves and assign it to the uncertainty in γ. We do this by equating the covariance matrix, COV, among the logarithm of the M Fusion model predictions across concentrations to the covariance of the approximate Fusion model predictions [8]. That is:

$$\text{COV} = \sigma^2 T_{\omega}^{\mathcal{F}_A\prime} T_{\omega}^{\mathcal{F}_A} \qquad \text{Eq. S6}$$

$$\hat{\sigma}^2 = \left(T_{\omega}^{\mathcal{F}_A} T_{\omega}^{\mathcal{F}_A\prime}\right)^{-1} T_{\omega}^{\mathcal{F}_A} \text{COV} T_{\omega}^{\mathcal{F}_A\prime} \left(T_{\omega}^{\mathcal{F}_A} T_{\omega}^{\mathcal{F}_A\prime}\right)^{-1} \qquad \text{Eq. S7}$$

The uncertainty in the Fusion model predictions is larger when $z > \theta$ compared to when $z < \theta$. Model predictions are largely governed by the uncertainty in $\hat{\gamma}$ when $z < \theta$. However, the Fusion model uncertainty is governed by two parameter estimates, $(\hat{\gamma}, \hat{\rho})$, when $z > \theta$, adding more uncertainty in model predictions.

We therefore suggest that an estimate of $\hat{\sigma}^2$ be based on concentrations $z < 2\theta$ and not the full range for three reasons. First, the restricted range incorporates the same number of concentrations above and below θ such that the estimate of $\hat{\sigma}^2$ is not unduly influenced by the (potentially) much larger number of concentrations $z > \theta$. Second, the restricted range does include concentrations from both parts of the model with different stochastic structures. Third, twice the cohort exposure range is likely to encompass most concentrations of interest in most benefits analyses.

The approximation specification is completed by randomly generating M realizations of

$$\mathcal{F}_A(z) = \exp\left(N(\hat{\gamma}, \hat{\sigma}) \times T_{\omega}^{\mathcal{F}_A}(z)\right) \qquad \text{Eq. S8}$$

a form that can be utilized by BenMAP.

Table S2. Parameters for the Approximation of the Fusion model in BenMAP

Parameter	Value
γ (β in Eq. 1 and Eq. 2)	0.009267
Standard error γ	0.001438
τ_1	127.276
τ_2	36.56594
τ_3	197.5335
τ_4	-108.337
τ_5	32.95614
η_1	29.81043
η_2	362.1602
η_3	9999.845
η_4	27.96906
η_5	68.20174

As described in the main text, the relative risk coefficient of 1.06 per 10 ppb O_3 exposure (95% CI: 1.03, 1.10) for respiratory-related mortality from O_3 was taken from a meta-analysis of COPD and O_3 exposure from the 2019 Global Burden of Disease[9]. The COPD relative risk coefficient derived from the GBD was not age restricted and is thus applied in this analysis to all respiratory-related mortality baseline incidence for all ages. If this analysis were alternatively restricted to ages >25 years, this would reduce the estimated O_3-related mortality in each country by between 0-2%, and by 6% for Nigeria. In this study, we also assume the same risk coefficient down to an exposure level of 0 ppbv (i.e., a theoretical minimum risk exposure level of 0 ppbv). In contrast to more traditional health burden analyses, this assumption is made to facilitate the development of the reduced form functions that are intended to quantify the additional risk associated with an increase in future warming (further discussed in Text S3). However, the 2019 GBD recently suggested a uniform distribution of the long-term O_3 TMREL between 29.1 and 35.7 ppbv, with a median of 32.4 ppbv. As shown in Figure S3, the global average MDA8O_3 in the baseline scenario is higher than this TMREL, with future O_3 reductions in populated areas small enough to keep the exposure levels during the O_3 season above this limit. However, of the 201 countries assessed in the analysis, 48 had a national average seasonal MDA8O_3 value of less than 32.4 ppbv in both the GISS and CESM-driven GEOS-Chem simulations in the baseline scenario. In the SSP5-8.5 scenario, 46 countries had a national average less than the TMREL in the CESM-driven GEOS-Chem simulations, while 52 countries had a national average below the TMREL in the GISS-driven simulations. Most of these are coastal countries or small island nations where increased water vapor in marine air leads to lower

concentrations of O_3 (Figure S2). As a sensitivity test to this selection of a 0 ppbv O_3 TMREL, we remove the change in deaths that are projected to occur in countries with O_3 concentrations <32.4 ppbv in each of the six future scenarios. This elimination reduces the global number of deaths by <1% in four of the six future scenarios, increases the global number of deaths by <1% in one scenario, and reduces the number of global deaths by 6% in one scenario (CESM-driven SSP1-2.6). This sensitivity test shows that while the choice of TMREL introduces source of uncertainty that may overestimate O_3 impacts in 2100 in select countries that are strongly influenced by marine air, this selection does not significantly change the global conclusions of this analysis and helps with the development of the impact-by-degree functions (see Supporting Information Text S3). This is also likely a smaller source of uncertainty than the additional uncertainties in the GCM and CTM modeling.

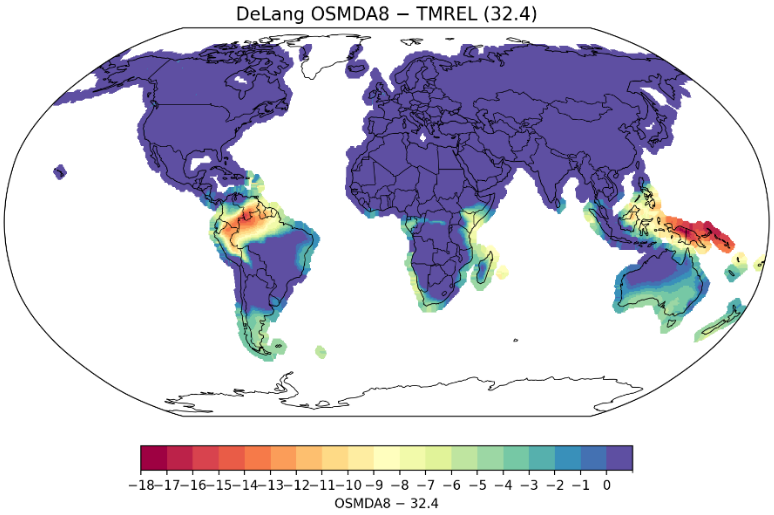

Figure S2. Locations with seasonal surface MDA8O_3 concentrations less than 32.4 ppbv in the downscaled base simulation (from DeLang et al., 2021). The color bar shows the magnitude (in ppbv) below 32.4 ppbv.

Supplemental Text S1. Ozone and $PM_{2.5}$ Concentrations

Figures S2 and S3 illustrate the absolute change in ozone season MDA8O_3 and annual average population-weighted $PM_{2.5}$ concentrations in the (bias corrected) CESM- and GISS-driven GEOS-Chem reference period simulations. Additional panels in these figures display the average global ΔMDA8O_3 and population-weighted $\Delta PM_{2.5}$, when GEOS-Chem is driven by the meteorological parameters for SSP1-2.6, SSP2-4.5, and SSP5-8.5 scenarios from the GISS and CESM GCMs.

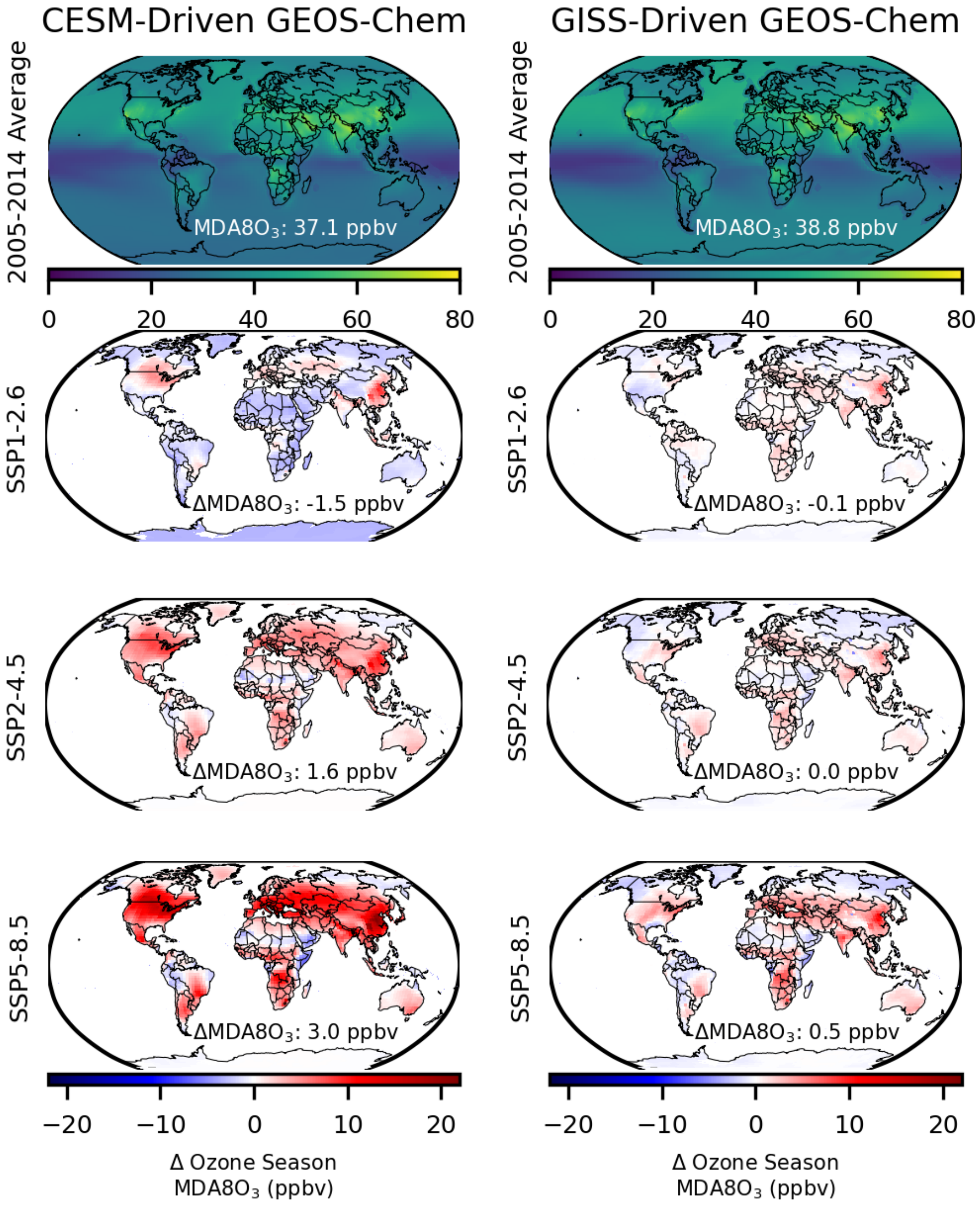

Figure S3. Global O_3 mixing ratios from the CESM (left) and GISS-driven (right) GEOS-Chem simulations. Top row) average $\Delta MDA8O_3$ over the 2005-2014 reference period. Bottom three rows) change in average $\Delta MDA8O_3$ in each 2090-2099 simulation relative to the reference period. The absolute (or change in) land-average $\Delta MDA8O_3$ is given in the bottom right of each panel.

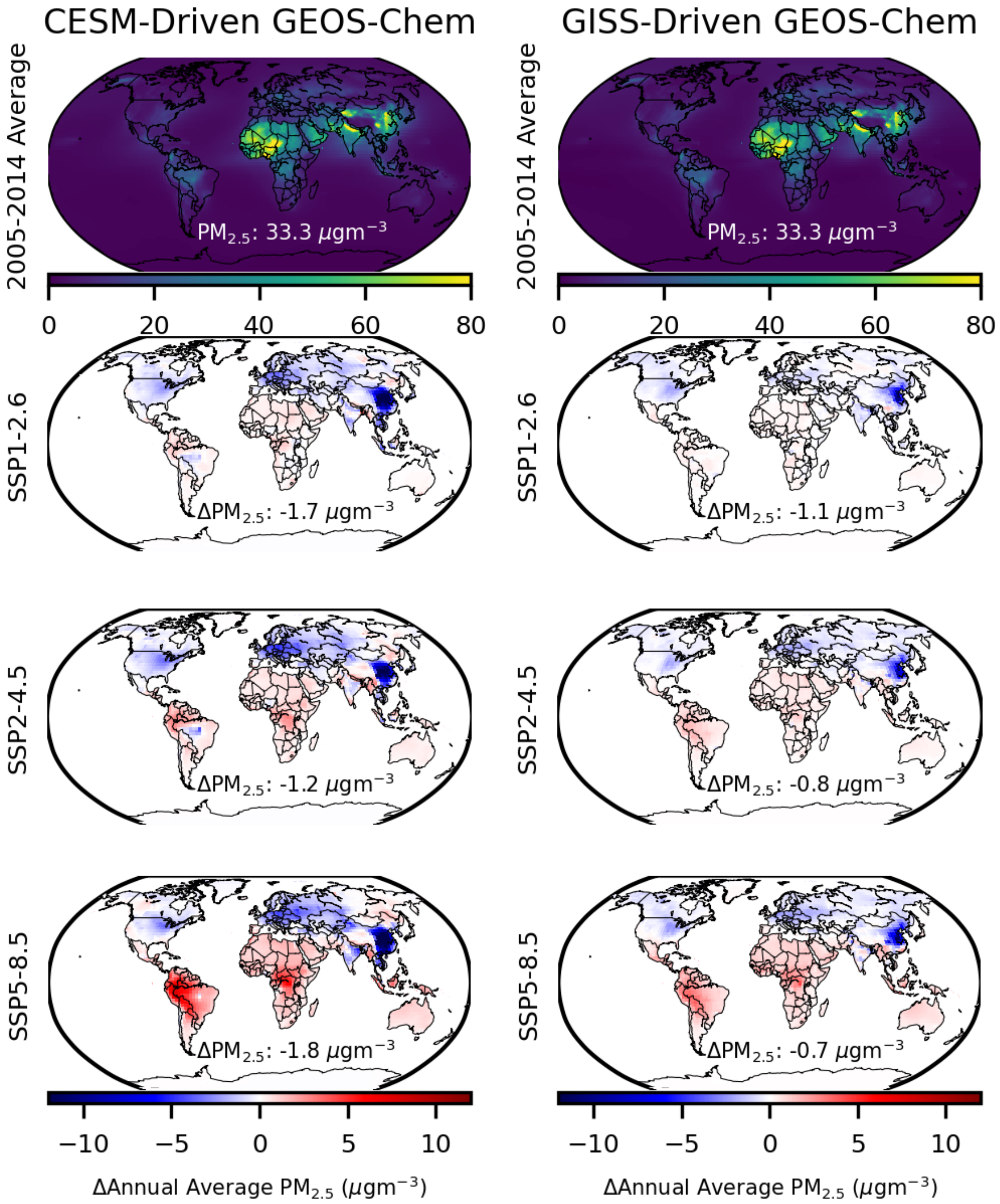

Figure S4. $PM_{2.5}$ results from the CESM (left) and GISS-driven (right) GEOS-Chem simulations. Top row) annual average $PM_{2.5}$ concentrations (including dust and salt) over the 2005-2014 reference period. Bottom three rows) change in annual average $PM_{2.5}$ concentration in the 2090s decade relative to the reference period. The absolute annual population-weighted average $PM_{2.5}$ concentration (or change in concentration) is given in the bottom right of each panel. For this calculation, the population data are held at 2020 values.

Table S3. Atmospheric processes that impact $\Delta MDA8O_3$

Process[a]	Simulated Trend[b]	Primary $MDA8O_3$ Impact[c]	Theoretical Impact Mechanism (additional references)
Temperature	+ (GISS)<br>+ (CESM)	+	a) higher temperatures decrease the PAN lifetime (NO_x + HO_x reservior), which can increase O_3 precursors and O_3 production<br>b) higher temperatures may increase BVOC emissions (discussed below), but may suppress emissions at the highest temperatures, depending on factors such as land cover type and soil moisture<br>c) higher temperatures are often correlated with stagnant conditions (see below) that increase O_3 production[10]
Tropospheric water vapor	+ (GISS)<br>+ (CESM)	+ or -	R1) $O_3 + hv \rightarrow O_2 + O(1D)$<br>R2) $H_2O + O(1D) \rightarrow OH$<br>R3) $O(1D) + O_2 + M \rightarrow O_3$<br>a) Water vapor increases the formation of OH (R2) rather than the regeneration of O_3 (R3). In less polluted environments (which are more influenced by background O_3), this will decrease background O_3 and regional O_3 concentrations<br>b) in polluted environments, increased OH production (R2) can increase the oxidation of CO and VOCs, which increase O_3 production; OH can react with NO_2 to form HNO_3, which can reduce the efficiency of O_3 production[11, 12]
Regional Stagnation[d]	+ (GISS)<br>+ (CESM)	+	Conditions characterized by a stable troposphere, including light winds, no precipitation, or temperature inversions can decrease the ventilation of O_3 and its precursors; inversely, the build-up of chemical concentrations can increase production[13]
Dry Deposition Velocity[e]	N/A (GISS)<br>+ (CESM)	-	Increased soil moisture or uptake by surface vegetation increases O_3 loss to the surface[14, 15]
Planetary Boundary Layer Height	+ (GISS)<br>+ (CESM)	+ or -	PBL expansion leads to dilution, but may also increase O_3 by mixing in more polluted background air or increasing the O_3 production efficiency[16]
Non-methane BVOCs	+ (GISS)<br>+ (CESM)	+ or -	Increased emissions of BVOCs may increase regional O_3 concentrations if O_3 production is VOC-limited, or decrease regional O_3 concentrations if O_3 production is VOC-saturated (or NO_x-limited)[10]
Lightning NO_x	+ (GISS)<br>+ (CESM)	+ or -	Increases in NO_x from lightning may increase O_3 concentrations if regional production is NO_x-limited, or decrease O_3 if regional production is NO_x-saturated; may also impact background O_3 concentrations[17]
Soil NO_x	+ (GISS)<br>+ (CESM)	+ or -	Increased NO_x emissions from temperature-dependent microbial activities in soils may increase O_3 if regional production is NO_x-limited, or decrease O_3 concentrations if regional production is NO_x-saturated[18]
Stratospheric O_3 transport	Not assessed	+	Intrusion of O_3-rich stratospheric air will increase O_3 concentrations in the troposphere[19]

[a] Does not include processes not considered in this modeling framework, including changes anthropogenic emissions, methane concentrations, or wildfires, which were all held constant across simulations

[b] defined as whether the ozone season globally averaged process (over land only) increased or decreased in the 2090s SSP2-4.5 GISS- and CESM-driven GEOS-Chem simulations relative to the reference period (2005-2014) simulation.

[c] Indicates whether an increase in each process will increase or decrease ozone season $MDA8O_3$

[d] There is no single model diagnostic parameter indicative of stagnant conditions in the troposphere. We consider decreases in horizontal winds, total precipitation, and an increase in the potential temperature difference between the top and bottom of the troposphere to be correlated with stagnant conditions. Atmospheric stability is generally expected to increase in the tropics and mid-latitudes due to increases in latent heat in the upper troposphere in the future, while atmospheric stability over the polar regions is expected due to increases in surface warming.

[e] dry deposition velocities were not archived from the 2090s GISS-driven GEOS-Chem simulations

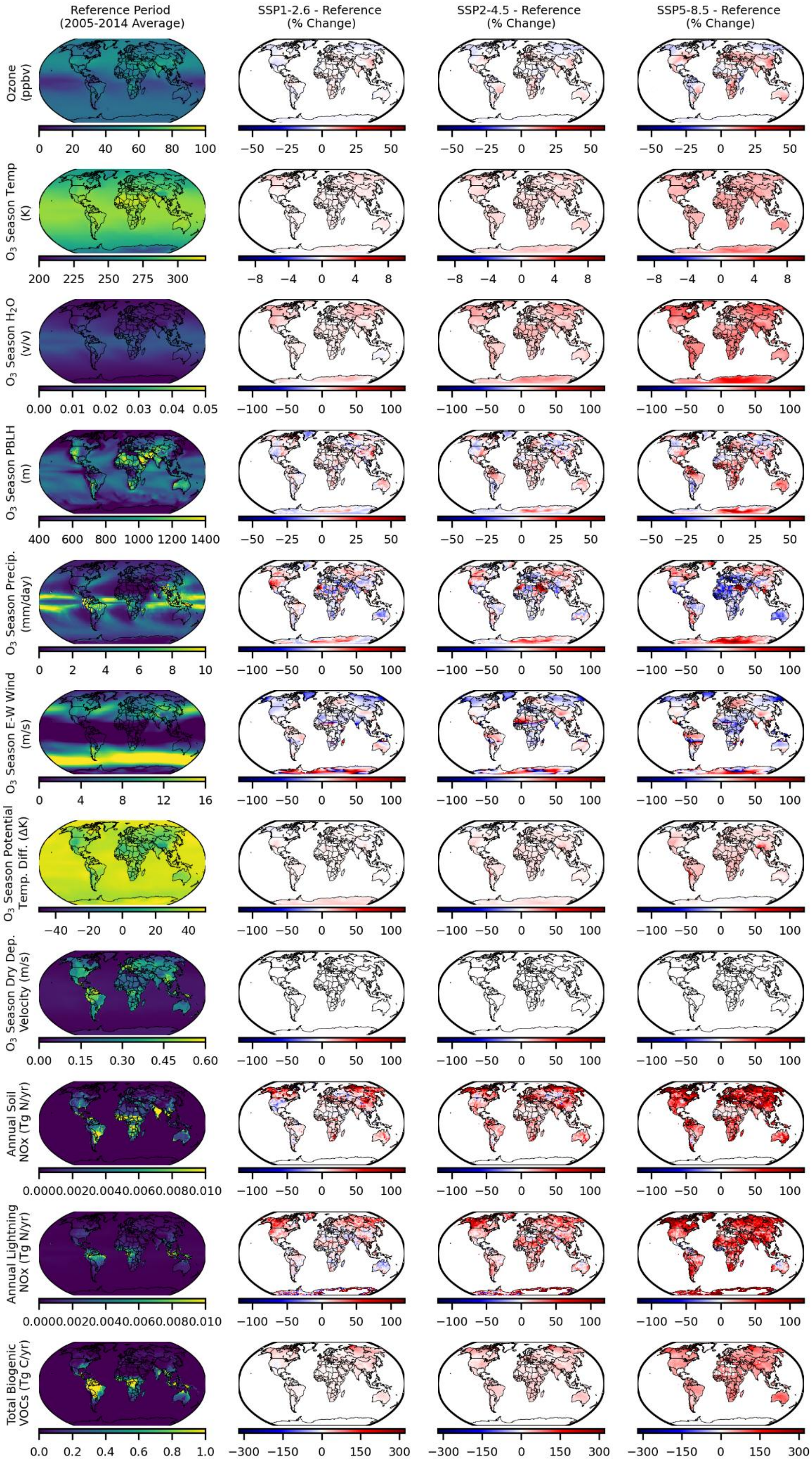
Reference Period (2005-2014 Average)
SSP1-2.6 - Reference (% Change)
SSP2-4.5 - Reference (% Change)
SSP5-8.5 - Reference (% Change)
Ozone (ppbv)
O_3 Season Temp (K)
O_3 Season H_2O (v/v)
O_3 Season PBLH (m)
O_3 Season Precip. (mm/day)
O_3 Season E-W Wind (m/s)
O_3 Season Potential Temp. Diff. (ΔK)
O_3 Season Dry Dep. Velocity (m/s)
Annual Soil NOx (Tg N/yr)
Annual Lightning NOx (Tg N/yr)
Total Biogenic VOCs (Tg C/yr)
0 20 40 60 80 100
−50 −25 0 25 50
200 225 250 275 300
−8 −4 0 4 8
0.00 0.01 0.02 0.03 0.04 0.05
−100 −50 0 50 100
400 600 800 1000 1200 1400
0 2 4 6 8 10
0 4 8 12 16
−40 −20 0 20 40
0.00 0.15 0.30 0.45 0.60
0.0000.0020.0040.0060.0080.010
0.0 0.2 0.4 0.6 0.8 1.0
−300 −150 0 150 300

Figure S5. Trends in select ΔMDA8O_3, meteorological parameters, and natural NO_x and VOC emissions from the GISS-driven GEOS-Chem simulations. The first column shows the absolute value of each metric in the reference simulation, while columns 2-4 illustrate the percent change in each metric as calculated by: 100* (SSP scenario results – reference scenario results)/reference scenario results. Ozone season results were calculated by taking the average of the 6 months with the highest O_3 concentrations in the Northern and Southern Hemisphere. The emission values are total annual emissions. Note that the GISS-driven meteorological data do not include dry deposition diagnostics.

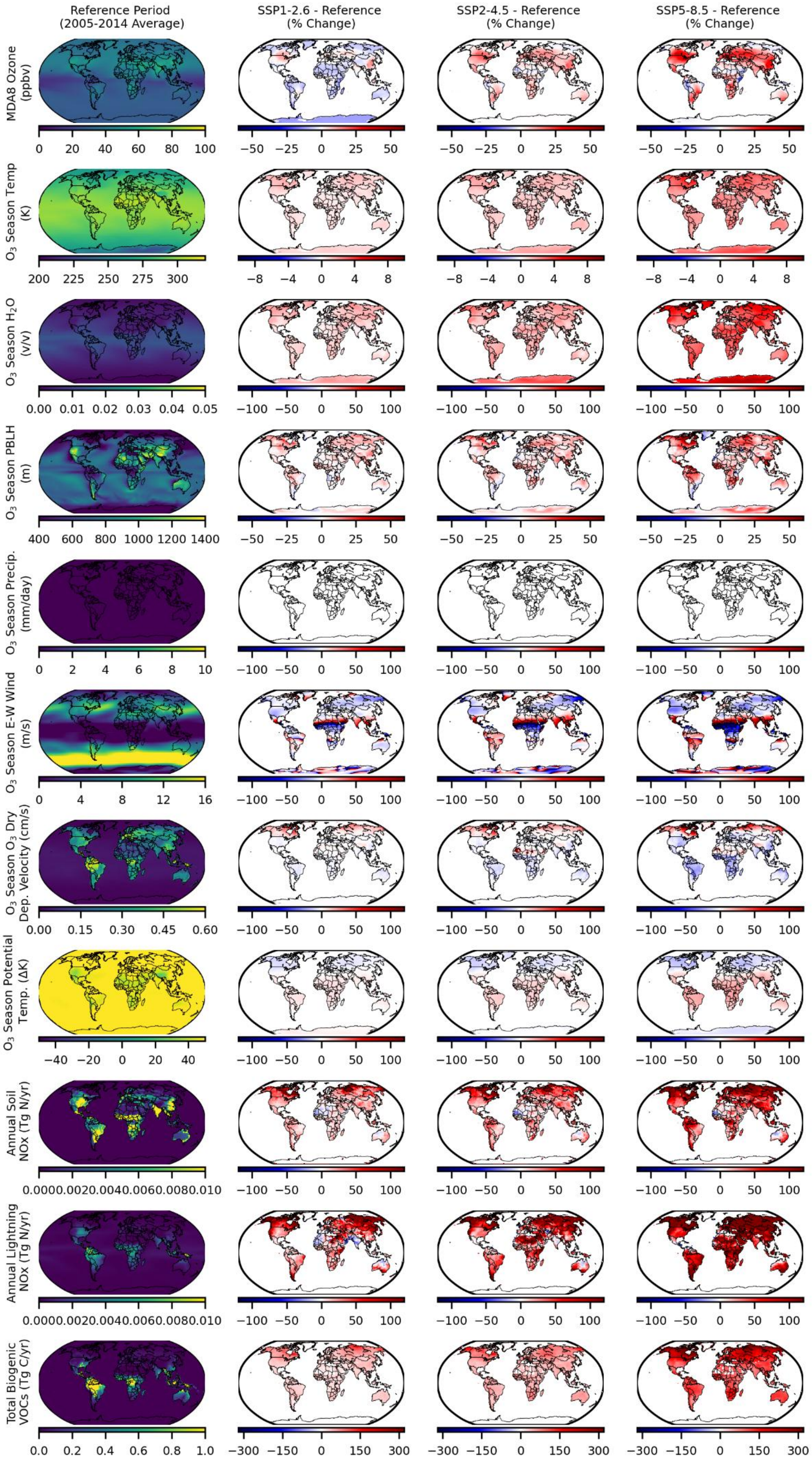

Reference Period (2005-2014 Average)
SSP1-2.6 - Reference (% Change)
SSP2-4.5 - Reference (% Change)
SSP5-8.5 - Reference (% Change)
MDA8 Ozone (ppbv)
O3 Season Temp (K)
O3 Season H2O (v/v)
O3 Season PBLH (m)
O3 Season Precip. (mm/day)
O3 Season E-W Wind (m/s)
O3 Season O3 Dry Dep. Velocity (cm/s)
O3 Season Potential Temp. (ΔK)
Annual Soil NOx (Tg N/yr)
Annual Lightning NOx (Tg N/yr)
Total Biogenic VOCs (Tg C/yr)

Figure S6. Same as Figure S5, but for CESM-driven GEOS-Chem simulations. Note that the CESM-driven meteorological data do not include total precipitation diagnostics.

Table S4. Atmospheric processes that impact annual $PM_{2.5}$ concentrations.

**Process**[a]	**Simulated Trend**[b]	**Primary $PM_{2.5}$ Impact**[c]	**Theoretical Impact Mechanism (additional references)**
Temperature	+ (GISS)<br>+ (CESM)	+ (SO_4, OA)<br>- (NIT, NH_4)	a) higher temperatures favor NH_4 and nitrate (NIT) thermodynamic partitioning to the gas phase<br>b) higher temperatures may increase BVOC emissions (discussed below), but may suppress emissions at the highest temperatures, depending on factors such as land cover type and soil moisture; higher temperatures increase volatilization which can decrease organic aerosol (OA)<br>c) higher temperatures often correlate with stagnant conditions (see below), increasing $PM_{2.5}$ production<br>d) higher temperatures increase SO_2 oxidation rates[11, 20]
Wet Deposition Rate[d]	N/A (GISS)<br>+ (CESM)	- (all)	Wet deposition is the primary loss pathway for $PM_{2.5}$. Stronger correlations have been observed with precipitation frequency rather than total precipitation[11, 21, 22]. The rate of wet deposition is dependent on the atmospheric concentration of $PM_{2.5}$
Dry Deposition Velocity[d]	N/A (GISS)<br>+ (CESM)	- (all)	Increased surface uptake of $PM_{2.5}$ components
Regional Stagnation[e]	Undetermined (GISS & CESM)	+ (all)	Conditions characterized by a stable troposphere, including light winds, no precipitation, or temperature inversions can decrease the ventilation of $PM_{2.5}$ and its precursors and increase $PM_{2.5}$ chemical production; inversely, increasing rain and wind can increase ventilation rates[21]
Planetary Boundary Layer Height	+ (GISS)<br>+ (CESM)	- (all)	PBL expansion leads to dilution and lower concentrations. (Background $PM_{2.5}$ concentrations are lower than O_3 and therefore mixing in background air leads to net lower concentrations compared to the effect on O_3)[21]
Tropospheric Water Vapor	+ (GISS)<br>+ (CESM)	+ (SO_4)	Increases in water vapor increase concentrations of SO_2 oxidants and increase the uptake of semi-volatile components[20]
Non-methane BVOCs	+ (GISS)<br>+ (CESM)	+ (OA)	Increased emissions of BVOCs can increase regional $PM_{2.5}$ concentrations through increased oxidation and condensation[23]
Lightning NO_x	+ (GISS)<br>+ (CESM)	+NIT, SO_4	Increased lightning increases aerosol nitrate in the upper troposphere (and may increase sulfate from increased SO_2 oxidation)[24]
Soil NO_x	+ (GISS)<br>+ (CESM)	+NIT, NH_4	Increased emissions can lead to increased HNO_3 production and NH_4NO_3 formation, dependent on environmental conditions

[a] This list does not include processes that were not considered in this modeling framework, including changes in anthropogenic emissions, dust, sea salt, or wildfires, which were all held constant across all simulations
[b] defined as whether the annual globally averaged process (over land only) increased or decreased in the 2090s SSP2-4.5 GISS- and CESM-driven GEOS-Chem simulations relative to the reference period (2005-2014) simulation.
[c] Indicates whether an increase in each process will increase or decrease annual average (land only) $PM_{2.5}$ and its components
[d] Dry and wet deposition diagnostics were not archived from the GISS-driven GEOS-Chem simulations
[e] See Table S3

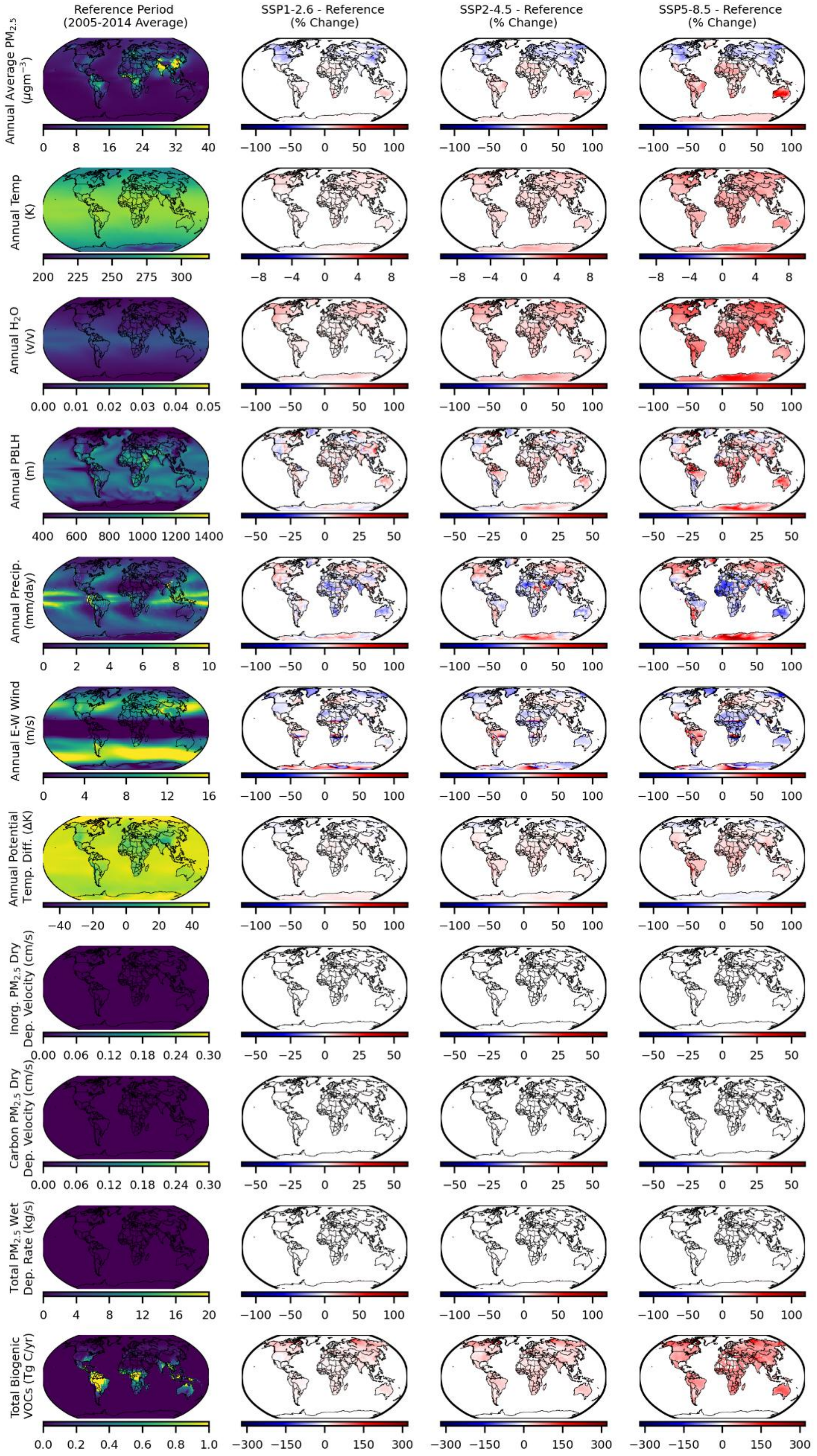

Reference Period (2005-2014 Average)
SSP1-2.6 - Reference (% Change)
SSP2-4.5 - Reference (% Change)
SSP5-8.5 - Reference (% Change)
Annual Average $PM_{2.5}$ (μgm^{-3})
Annual Temp (K)
Annual H_2O (v/v)
Annual PBLH (m)
Annual Precip. (mm/day)
Annual E-W Wind (m/s)
Annual Potential Temp. Diff. (ΔK)
Inorg. $PM_{2.5}$ Dry Dep. Velocity (cm/s)
Carbon $PM_{2.5}$ Dry Dep. Velocity (cm/s)
Total $PM_{2.5}$ Wet Dep. Rate (kg/s)
Total Biogenic VOCs (Tg C/yr)

Figure S7. Trends in select annual average $PM_{2.5}$ mass, meteorological parameters, dry deposition velocity, wet deposition rates, and VOC emissions from the GISS-driven GEOS-Chem simulations. GISS-driven data do not include deposition diagnostics. The first column shows the absolute value of each metric in the reference simulation; columns 2-4 illustrate the percent change in each metric as calculated by: 100* (SSP scenario results – reference scenario results)/reference scenario results.

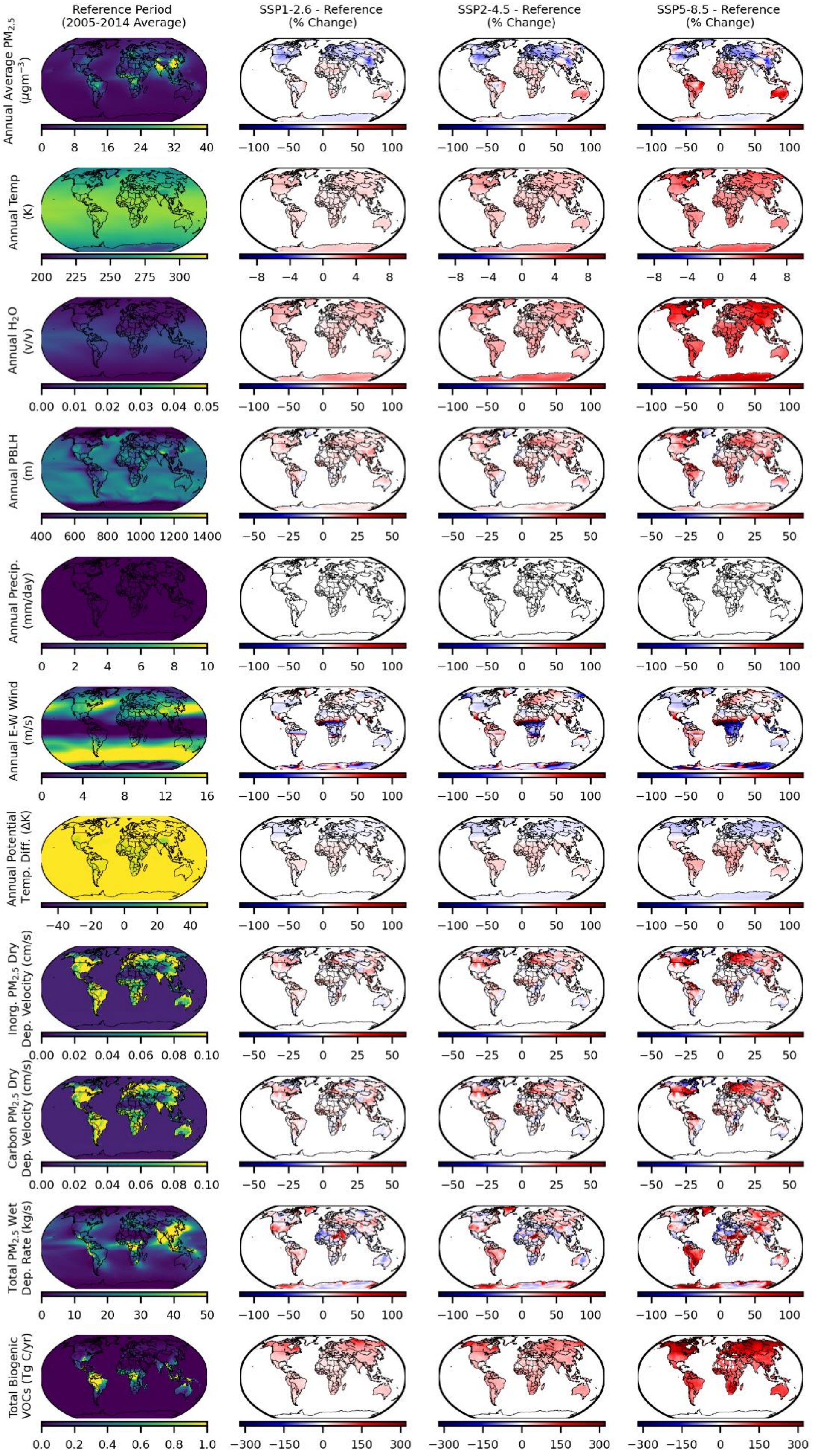
Reference Period
(2005-2014 Average)
SSP1-2.6 - Reference
(% Change)
SSP2-4.5 - Reference
(% Change)
SSP5-8.5 - Reference
(% Change)
Annual Average $PM_{2.5}$
(μgm^{-3})
Annual Temp
(K)
Annual H_2O
(v/v)
Annual PBLH
(m)
Annual Precip.
(mm/day)
Annual E-W Wind
(m/s)
Annual Potential
Temp. Diff. (ΔK)
Inorg. $PM_{2.5}$ Dry
Dep. Velocity (cm/s)
Carbon $PM_{2.5}$ Dry
Dep. Velocity (cm/s)
Total $PM_{2.5}$ Wet
Dep. Rate (kg/s)
Total Biogenic
VOCs (Tg C/yr)

Figure S8. Same as Figure S7, but for CESM-driven GEOS-Chem simulations. Note that the CESM-driven data do not include precipitation diagnostics.

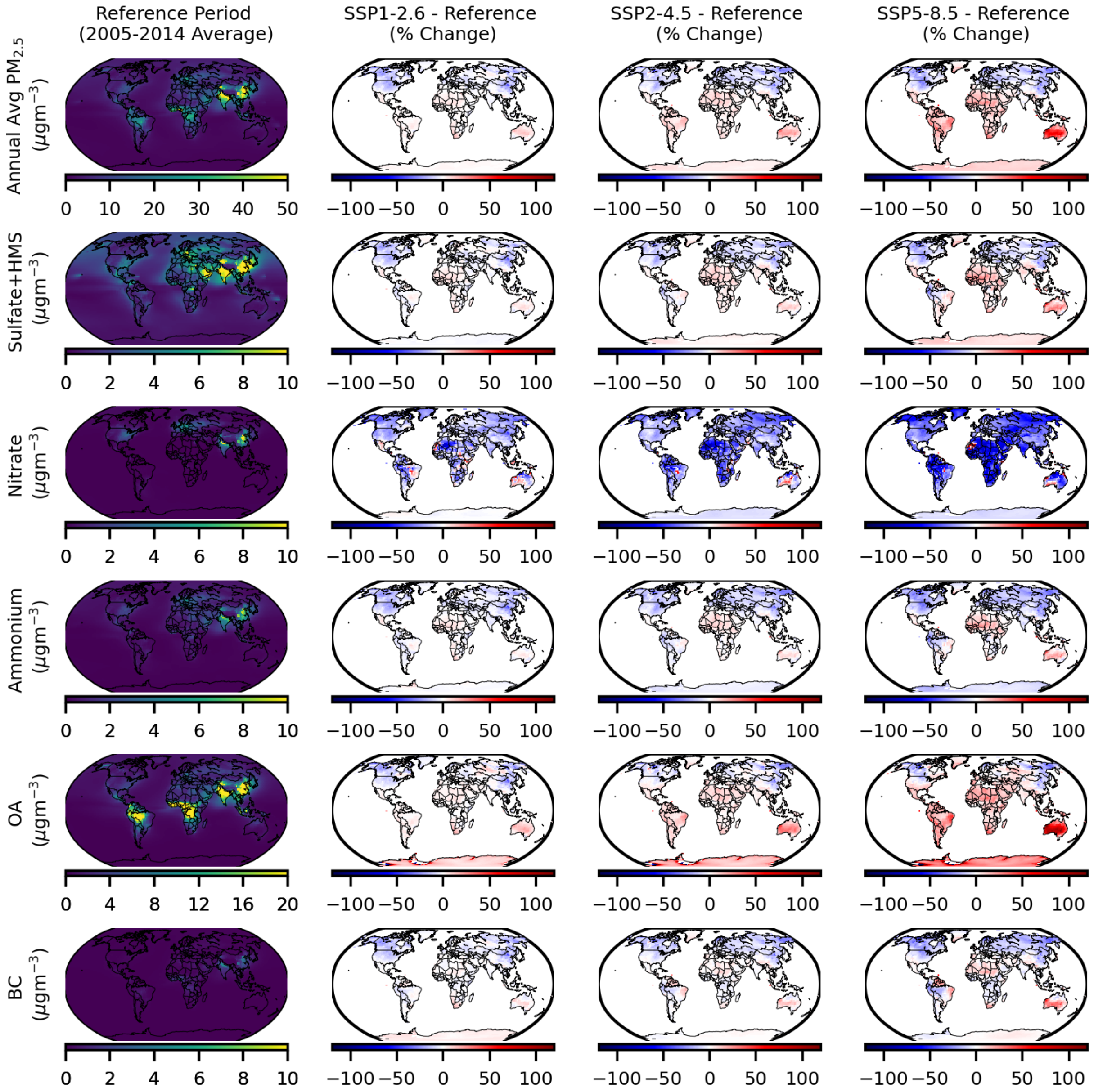

Figure S9. Trends in individual $PM_{2.5}$ components from the GISS-driven GEOS-Chem simulations. The first column shows the absolute value of each component in the reference simulation, while columns 2-4 illustrate the percent change in each component as calculated by: 100* (SSP scenario results – reference scenario results)/reference scenario results. Note the change in color ranges in the first column.

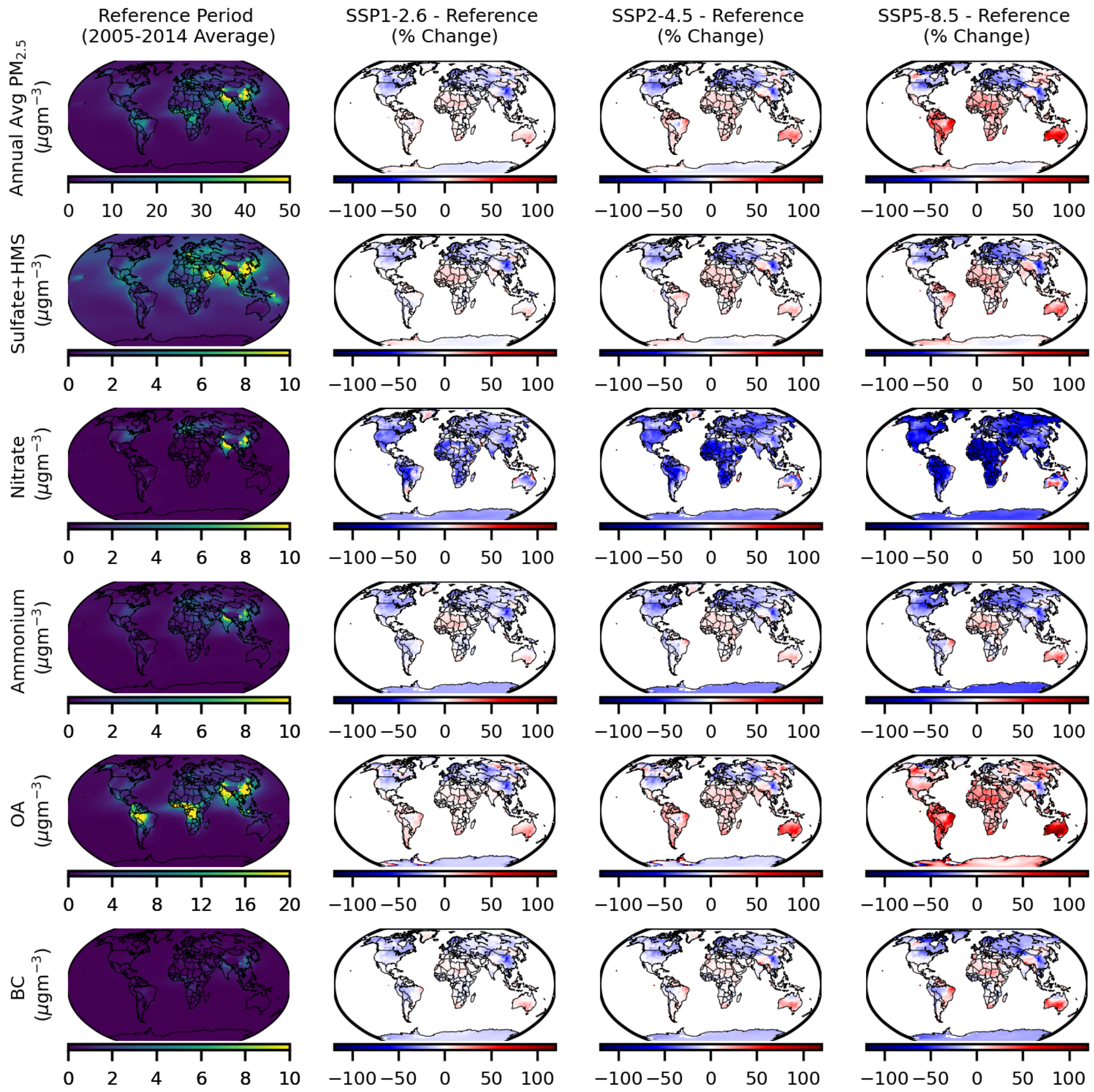

Figure S10. Same as Figure S9, but for CESM-driven GEOS-Chem simulations.

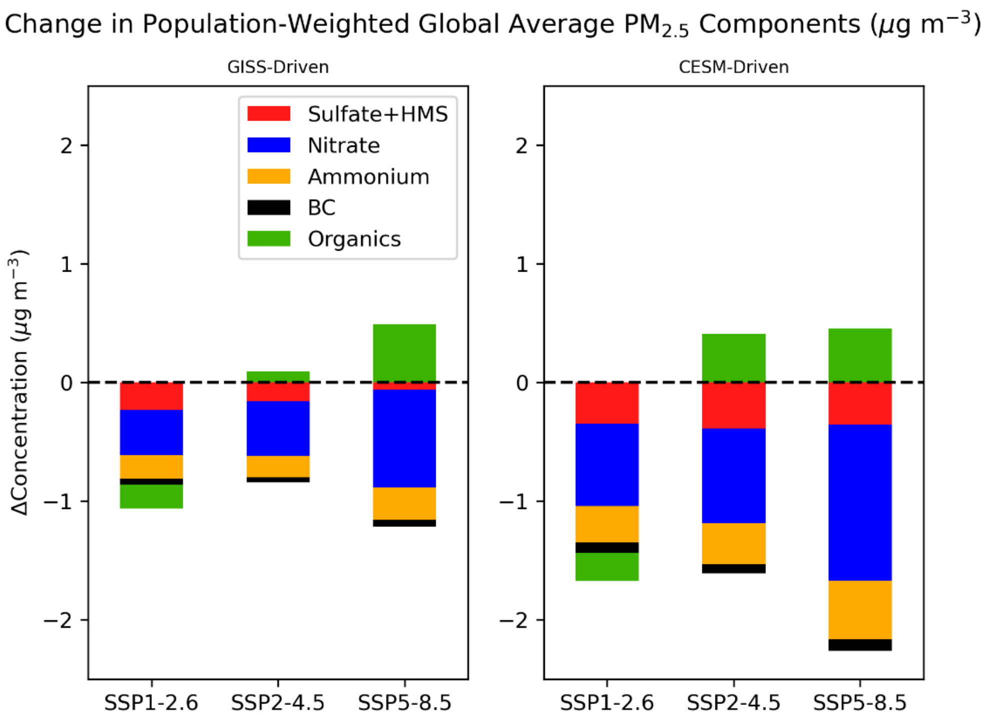

Figure S11. Change in globally averaged, population-weighted $PM_{2.5}$ components, by scenario.

Supporting Information Text S3. Impact-by-Degree Functions, Monetization, and Net Present Value

As described in the Main Text, the mortality per capita estimates for each of the six GCM-driven GEOS-Chem simulations are used to develop country-specific impact functions by degree of future warming. Indexing the dynamic and complex mortality changes to temperature in this way enables translation of six individual scenario-based results into time- and scenario-independent functions that can be applied to any future year or warming scenario. For example, as illustrated in Figures 3 and 4, we develop two impact functions for each country, from the per capita impacts at the global temperatures in each of the six GEOS-Chem scenarios (3 each for GISS and CESM-driven simulations), linearly interpolating between each point. To estimate impacts in cases where future temperature may exceed those in the SSP5-8.5 scenario (as can be predicted by other GCMs or in years after 2100), the functions are held constant at the per capita impacts and their warmest value (>5°C for CESM-informed functions and 3.14°C for GISS-informed functions).

While the reduced form tool takes total population as input (not just the age ranges relevant to the exposure metrics), the functions are developed using the number of deaths per capita for each country, as calculated for each future scenario using the age-specific population inputs and impact functions in BenMAP. This analysis reports BenMAP results using the population characteristics from the 2090's

decade in order to capture the potential impact of an aging population. Therefore, the reduced form tool will assume this same population distribution each year. This represents an additional source of uncertainty in the reduced form tool as the age structure of the population will likely not remain constant over the following centuries.

In addition, as described in the Main Text and Text S2, the reduced form temperature impact functions are developed from estimates of deaths per capita in each country at all levels of O_3 and $PM_{2.5}$ exposure (i.e., assuming a TMREL of 0). While recent epidemiological studies support a TMREL of 0 $\mu g/m^3$ for $PM_{2.5}$, the latest evidence considered in the GBD supports a TMREL between 29.1-35.7 ppbv (32.4 ppbv central value) for O_3. The sensitivity test in Text S2 shows that because most of the global population is exposed to $MDA8O_3$ concentrations higher than this value, implementing the O_3 TMREL would only change global mortality estimates in 2100 by <1% in five of the six simulations used to develop the impact functions (though changes can be up to 100% in select individual countries). Implementing a non-zero TMREL in the impact functions, however, would introduce discontinuities and additional uncertainties in the impact functions for countries at or near the TMREL. For example, $MDA8O_3$ concentrations in Bolivia are >32.4 ppbv in the base simulation, drop to between 30.2 and 32.4 ppbv in the SSP1-2.6 and SSP5-8.5 scenarios, but are >32.4 in the SSP2-4.5 scenario. This would result in zero damages per capita in Bolivia at all temperatures other than between 0.62 - 3.18°C in the GISS-informed functions and between 1.42- 5°C in the CESM-informed functions, which may not reflect actual damages (in the case where the actual exposure threshold is not exactly 32.4 ppbv). This example also demonstrates another source of uncertainty that could be introduced at higher temperatures. For example, in the absence of additional information, the impact functions at high temperatures are held constant at the number of deaths per capita from the highest temperature simulation (SSP5-8.5 scenario). Therefore, in cases where damages are zero in the SSP5-8.5 scenario when implementing a non-zero TMREL, the functions would then assume a constant impact of 0 deaths per capita at all higher temperatures, regardless of the corresponding change in O_3 concentration. This assumption could underestimate impacts in countries where concentrations cross the TMREL threshold at higher (>3.14° or 5°C) temperatures, or overestimate impacts in countries where O_3 concentrations cross under the TMREL at these higher temperatures. Therefore, due to the small sensitivity of the global results and to be consistent with previous similar studies[25], we chose to minimize discontinuities in the temperature damage functions in this analysis by implementing a 0 ppbv TMREL. However, this does highlight caution in interpreting O_3 impacts or applying impact functions to select countries in Oceania, Southeast Asia, and Latin America (Figure S2).

Monetization of the health impacts and calculation of the NPV are discussed in detail in the remainder of this section. As in previous analyses[25], the VSL refers to an individual's willingness to pay (averaged across the population for each country) for a small reduction in the risk of their own premature death within each future year, and does not consider non-mortality-related costs, such as environmental effects on labor productivity or direct spending on health care. The VSL is not intended to reflect the price of an individual life but is an aggregate sum that individuals are willing to pay to reduce the risk of premature death. In countries where meteorological and emission-driven changes are projected to increase air pollution, the VSL is used to monetize the damages associated with the increased levels of exposure and mortality. Conversely, in countries where these same meteorological driven changes are projected to decrease exposure to air pollution (e.g., increased precipitation reducing in $PM_{2.5}$), the VSL is used to monetize the benefits of the number of avoided premature deaths. The VSL is year and country-specific, as calculated by Eq. S9.

$$\mathrm{VSL}_{c,t} = \mathrm{VSL}_{\mathrm{US},2024} \times \left(\frac{\mathrm{Income}_{c,t}}{\mathrm{Income}_{\mathrm{US},2024}}\right)^{\varepsilon} \qquad \text{Eq. S9}$$

The VSL for each country (c) and year (t) is calculated by scaling the U.S. VSL by the ratio of income in each country in each year. $\mathrm{VSL}_{\mathrm{US},2024}$ is the EPA 1990 value of the VSL for the U.S.[26], adjusted for income growth and inflation to \$13.9 million in 2024 dollars[27]. $\mathcal{E}$ is the income elasticity and is set to 1, such that the VSL is proportional to income in each country[28, 29]. As with previous studies, we approximate future changes in income as GDP per capita. Using this approach and the projections GDP and population for each country from the RFF-SP scenarios, the VSL in each country is, on average, is estimated to be between 4 to 15 times larger in 2100 than in 2024. We note that monetizing physical mortality using the VSL is an active area of research[30] and represents a source of uncertainty in this study.

To monetize the impacts in the year 2100, the change in the number of air pollution related deaths in 2100 relative to the reference period (2005-2014) is first calculated based on the global temperature change and national-level population in each country in the year 2100 in each of the 10,000 RFF-SP scenarios[31, 32]. This change in the number of deaths is then multiplied by the country-specific VSL to monetize impacts in the year 2100 in each country. Our central estimates presented in the main text are the sum of the impacts across 201 countries worldwide, averaged across all 10,000 probabilistic future scenarios. All results in this analysis are presented in 2024 US dollars.

To subsequently calculate the Net Present Value (NPV) of these impacts, the full stream of monetized annual O_3 and $PM_{2.5}$ mortality impacts for each country in the years 2030 through 2300, are discounted back to the year 2030 and integrated over time, as shown in Eq S10.

$$\text{NPV}_c = \sum_{t=2030}^{t=2300} \frac{\text{Annual damages}_{c,t}}{\prod_{x=2030}^{x=t}(1+ \text{Ramsey discount factor}_{c,t})} \qquad \text{Eq. S10}$$

The Ramsey discount factor is calculated in Eq S11.

$$\text{Ramsey discounting factor}_{c,t} = \rho + \eta g_{c,t} \qquad \text{Eq. S11}$$

where $(g_{c,t})$ is per capita economic consumption growth in each country from the year of the emissions pulse to year t, ρ is the pure rate of time preference, and η is the elasticity of the marginal value of consumption with change in $g_{c,t}$. The process of discounting converts future impacts into present dollar equivalents, which accounts for the fact that an additional dollar in the future will typically be valued less than an additional dollar in the present. We use factors that are calibrated to a near-term discount rate of 2.0%, which allows the discount rate to scale over time with future economic growth, so that an additional dollar is more highly valued in future scenarios with low economic growth. This analysis uses country specific discount factors, based on country-level consumption growth rather than the world average, which results in more conservative NPV estimates, but is consistent with the country-specific VSL calculation.

For the NPV calculation, we use 10,000 probabilistic RFF-SP scenarios of global temperature, population, and GDP from 2030 through 2300, with and without a pulse of CO_2 emissions in the year 2030 as inputs to the reduced form model. The temperature projections associated with the RFF-SP emissions are derived from FaIR (v1.6.2)[33], as developed for previous NPV estimates[34]. For context, the population and GDP growth rate trajectories are provided in Figures 3 and 6 of Rennert et al., 2021[35], and show an average population of roughly 11 billion by 2100 and a global GDP growth rate of roughly 1.5%. These values fall within the ranges estimated by the various SSP scenarios. These assumptions are still considered a relatively small uncertainty compared to additional uncertainties in future total population, income, and meteorological conditions. By developing a reduced form tool that aggregates the individual monetized health impacts from $PM_{2.5}$ and O_3 across all countries in this way, we provide a traceable estimate of the monetized damages from meteorologically driven changes in air pollution resulting from a marginal change in greenhouse gas emissions.

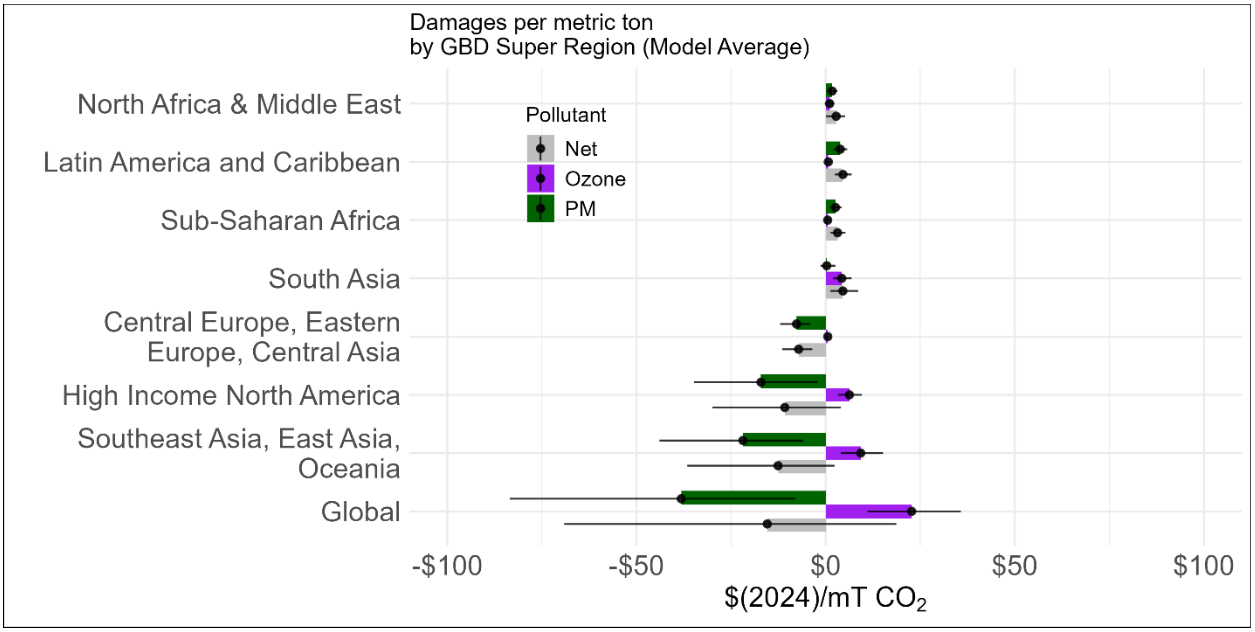

Figure S12. Impact per ton of CO_2 emissions for meteorological driven $PM_{2.5}$ and O_3-related mortality, presented by GBD super region. Values are calculated for a pulse of CO_2 emissions in the year 2030 and a 2% Ramsey discount rate. Error bars represent the mean and 75% CI of the 10,000 future RFF-SP projections.

Table S5. Percent change in the global deaths in the year 2100 compared to the global mean. The Concentration Response Function (CRF) row represents the change in mortality given the 95% confidence interval for the O_3 and $PM_{2.5}$ health functions, the GCM row represents the change in mortality given the meteorology inputs from the two GCMs relative to the multi-model-mean, and the population and temperature projection rows represent the change in mortality of the 2.5th and 97.5th percentiles across the 10,000 probabilistic RFF-SP scenarios, varying temperature only (holding population counts at the mean), population only (holding temperature at the mean), and a combination of changes in both population and temperature.

**Component/ Factor**[a]		**$PM_{2.5}$**	**O_3**	**Net**[b]
CRF	2.5th	31% fewer (benefit)	57% fewer (damage)	N/A*
	97.5th	31% more (benefit)	57% more (damage)	N/A*
GCM	CESM	38% fewer (benefit)	19% more (damage)	63% fewer (benefit)
	GISS	38% more (benefit)	19% fewer (damage)	63% more (benefit)
Pop. and Temperature Projections	2.5th	49% fewer (benefit)	68% fewer (damage)	156% fewer (**benefit**)
	97.5th	50% more (benefit)	111% more (damage)	200% more (**damage**)
Population only	2.5th	15% fewer (benefit)	9% fewer (damage)	25% fewer (benefit)
	97.5th	13% more (benefit)	9% more (damage)	21% more (benefit)
Temperature only	2.5th	46% fewer (benefit)	68% fewer (damage)	150% fewer (**benefit**)
	97.5th	49% more (benefit)	110% more (damage)	196% more (**damage**)

[a] Percent change in deaths listed in each column, with the sign of the impact given in parentheses. For example, the 97.5th percentile of the CRF results in a higher number of $PM_{2.5}$-related deaths than the mean (31% more), but with a net reduction (benefit) in the number of total $PM_{2.5}$-related deaths in 2100 compared to the 2005-2014 average. For O_3, the 97.5th percentile of the CRF results in a higher number of O_3-related deaths than the mean (57% more), but with a net increase (damage) in the number of total O_3-related deaths in 2100 compared to the 2005-2014 average.
[b] Net deaths are not estimated for the CRF because the individual O_3 and $PM_{2.5}$ CRFs may not be correlated with each other. For example, the upper estimate for the $PM_{2.5}$ CRF may not correspond to the upper estimate of the O_3 CRF. The net impacts in bold indicate a difference in trend between the upper and lower bounds of the sensitivity test (i.e., impacts are net benefits in one case and net damages in the other). The percent change from the individual $PM_{2.5}$ and O_3 results do not sum to the net impacts percent change because the net impacts are calculated as the sum of $PM_{2.5}$ and O_3 before the statistics are calculated across all 10,000 trials.

REFERENCES

1. Murray, L. T.; Leibensperger, E. M.; Mickley, L. J.; Tai, A. P. K., Estimating future climate change impacts on human mortality and crop yields via air pollution. *Proceedings of the National Academy of Sciences* **2024,** *121* (39), e2400117121.

2. Pai, S. J.; Heald, C. L.; Pierce, J. R.; Farina, S. C.; Marais, E. A.; Jimenez, J. L.; Campuzano-Jost, P.; Nault, B. A.; Middlebrook, A. M.; Coe, H.; Shilling, J. E.; Bahreini, R.; Dingle, J. H.; Vu, K., An evaluation of global organic aerosol schemes using airborne observations. *Atmos. Chem. Phys.* **2020,** *20* (5), 2637-2665.

3. Feng, L.; Smith, S. J.; Braun, C.; Crippa, M.; Gidden, M. J.; Hoesly, R.; Klimont, Z.; van Marle, M.; van den Berg, M.; van der Werf, G. R., The generation of gridded emissions data for CMIP6. *Geosci. Model Dev.* **2020,** *13* (2), 461-482.

4. Turnock, S. T.; Allen, R. J.; Andrews, M.; Bauer, S. E.; Deushi, M.; Emmons, L.; Good, P.; Horowitz, L.; John, J. G.; Michou, M.; Nabat, P.; Naik, V.; Neubauer, D.; O'Connor, F. M.; Olivié, D.; Oshima, N.; Schulz, M.; Sellar, A.; Shim, S.; Takemura, T.; Tilmes, S.; Tsigaridis, K.; Wu, T.; Zhang, J., Historical and future changes in air pollutants from CMIP6 models. *Atmos. Chem. Phys.* **2020,** *20* (23), 14547-14579.

5. DeLang, M. N.; Becker, J. S.; Chang, K.-L.; Serre, M. L.; Cooper, O. R.; Schultz, M. G.; Schröder, S.; Lu, X.; Zhang, L.; Deushi, M.; Josse, B.; Keller, C. A.; Lamarque, J.-F.; Lin, M.; Liu, J.; Marécal, V.; Strode, S. A.; Sudo, K.; Tilmes, S.; Zhang, L.; Cleland, S. E.; Collins, E. L.; Brauer, M.; West, J. J., Mapping Yearly Fine Resolution Global Surface Ozone through the Bayesian Maximum Entropy Data Fusion of Observations and Model Output for 1990–2017. *Environmental Science & Technology* **2021,** *55* (8), 4389-4398.

6. Hammer, M. S.; van Donkelaar, A.; Li, C.; Lyapustin, A.; Sayer, A. M.; Hsu, N. C.; Levy, R. C.; Garay, M. J.; Kalashnikova, O. V.; Kahn, R. A.; Brauer, M.; Apte, J. S.; Henze, D. K.; Zhang, L.; Zhang, Q.; Ford, B.; Pierce, J. R.; Martin, R. V., Global Estimates and Long-Term Trends of Fine Particulate Matter Concentrations (1998–2018). *Environmental Science & Technology* **2020,** *54* (13), 7879-7890.

7. Burnett, R. T.; Spadaro, J. V.; Garcia, G. R.; Pope, C. A., Designing health impact functions to assess marginal changes in outdoor fine particulate matter. *Environmental Research* **2022,** *204*, 112245.

8. Burnett, R.; Cork, M.; Fann, N.; Chen, H.; Weichenthal, S., Adapting non-parametric spline representations of outdoor air pollution health effects associations for use in public health benefits assessment. *Air Quality, Atmosphere & Health* **2024,** *17* (6), 1295-1305.

9. GBD 2019 Risk Factor Collaborators, Global burden of 87 risk factors in 204 countries and territories, 1990–2019: a systematic analysis for the Global Burden of Disease Study 2019. *The Lancet* **2020,** *396* (10258), 1223-1249.

10. Porter, W. C.; Heald, C. L., The mechanisms and meteorological drivers of the summertime ozone–temperature relationship. *Atmos. Chem. Phys.* **2019,** *19* (21), 13367-13381.

11. Jacob, D. J.; Winner, D. A., Effect of climate change on air quality. *Atmospheric Environment* **2009,** *43* (1), 51-63.

12. Johnson, C. E.; Collins, W. J.; Stevenson, D. S.; Derwent, R. G., Relative roles of climate and emissions changes on future tropospheric oxidant concentrations. *Journal of Geophysical Research: Atmospheres* **1999,** *104* (D15), 18631-18645.

13. Porter, W. C.; Heald, C. L.; Cooley, D.; Russell, B., Investigating the observed sensitivities of air-quality extremes to meteorological drivers via quantile regression. *Atmos. Chem. Phys.* **2015,** *15* (18), 10349-10366.

14. Clifton, O. E.; Paulot, F.; Fiore, A. M.; Horowitz, L. W.; Correa, G.; Baublitz, C. B.; Fares, S.; Goded, I.; Goldstein, A. H.; Gruening, C.; Hogg, A. J.; Loubet, B.; Mammarella, I.; Munger, J. W.; Neil, L.; Stella, P.; Uddling, J.; Vesala, T.; Weng, E., Influence of Dynamic Ozone Dry Deposition on Ozone Pollution. *Journal of Geophysical Research: Atmospheres* **2020,** *125* (8), e2020JD032398.

15. Pommier, M.; Fagerli, H.; Gauss, M.; Simpson, D.; Sharma, S.; Sinha, V.; Ghude, S. D.; Landgren, O.; Nyiri, A.; Wind, P., Impact of regional climate change and future emission scenarios on surface O3 and PM2.5 over India. *Atmos. Chem. Phys.* **2018,** *18* (1), 103-127.

16. Dawson, J. P.; Adams, P. J.; Pandis, S. N., Sensitivity of ozone to summertime climate in the eastern USA: A modeling case study. *Atmospheric Environment* **2007,** *41* (7), 1494-1511.

17. Murray, L. T., Lightning NOxand Impacts on Air Quality. *Current Pollution Reports* **2016,** *2* (2), 115-133.

18. Romer, P. S.; Duffey, K. C.; Wooldridge, P. J.; Edgerton, E.; Baumann, K.; Feiner, P. A.; Miller, D. O.; Brune, W. H.; Koss, A. R.; de Gouw, J. A.; Misztal, P. K.; Goldstein, A. H.; Cohen, R. C., Effects of temperature-dependent NOx emissions on continental ozone production. *Atmos. Chem. Phys.* **2018,** *18* (4), 2601-2614.

19. Meul, S.; Langematz, U.; Kröger, P.; Oberländer-Hayn, S.; Jöckel, P., Future changes in the stratosphere-to-troposphere ozone mass flux and the contribution from climate change and ozone recovery. *Atmos. Chem. Phys.* **2018,** *18* (10), 7721-7738.

20. Liao, H.; Chen, W.-T.; Seinfeld, J. H., Role of climate change in global predictions of future tropospheric ozone and aerosols. *Journal of Geophysical Research: Atmospheres* **2006,** *111* (D12).

21. Dawson, J. P.; Adams, P. J.; Pandis, S. N., Sensitivity of PM_{2.5} to climate in the Eastern US: a modeling case study. *Atmos. Chem. Phys.* **2007,** *7* (16), 4295-4309.

22. Xia, W.; Wang, Y.; Zhang, G. J.; Wang, B., Light Precipitation rather than Total Precipitation Determines Aerosol Wet Removal. *Environmental Science & Technology* **2024,** *58* (43), 19222-19230.

23. Heald, C. L.; Henze, D. K.; Horowitz, L. W.; Feddema, J.; Lamarque, J. F.; Guenther, A.; Hess, P. G.; Vitt, F.; Seinfeld, J. H.; Goldstein, A. H.; Fung, I., Predicted change in global secondary organic aerosol concentrations in response to future climate, emissions, and land use change. *Journal of Geophysical Research: Atmospheres* **2008,** *113* (D5).

24. Wang, H.; Pei, Y.; Yin, Y.; Shen, L.; Chen, K.; Shi, Z.; Chen, J., Observational Evidence of Lightning-Generated Ultrafine Aerosols. *Geophysical Research Letters* **2021,** *48* (14), e2021GL093771.

25. McDuffie, E. E.; Sarofim, M. C.; Raich, W.; Jackson, M.; Roman, H.; Seltzer, K.; Henderson, B. H.; Shindell, D. T.; Collins, M.; Anderton, J.; Barr, S.; Fann, N., The Social Cost of Ozone-Related Mortality Impacts From Methane Emissions. *Earth's Future* **2023,** *11* (9), e2023EF003853.

26. U.S. EPA, Guidelines for preparing economic analyses. Appendix B. Retrieved from https://www.epa.gov/environmental-economics/guidelines-preparing-economic-analyses. **2010**.

27. U.S. EPA, Supplementary material for the regulatory impact analysis for the supplemental proposed rulemaking, "Standards of performance for new, reconstructed, and modified sources and emissions guidelines for existing sources: Oil and natural gas sector climate review", EPA report on the social cost of greenhouse gases: Estimates incorporating recent scientific advances. Retrieved from: https://www.epa.gov/system/files/documents/2023-12/epa_scghg_2023_report_final.pdf. **2023**.

28. Hammitt, J. K.; Robinson, L. A., The Income Elasticity of the Value per Statistical Life: Transferring Estimates between High and Low Income Populations. *Journal of Benefit-Cost Analysis* **2011,** *2* (1), 1-29.

29. Rennert, K.; Errickson, F.; Prest, B. C.; Rennels, L.; Newell, R. G.; Pizer, W.; Kingdon, C.; Wingenroth, J.; Cooke, R.; Parthum, B.; Smith, D.; Cromar, K.; Diaz, D.; Moore, F. C.; Müller, U. K.; Plevin, R. J.; Raftery, A. E.; Ševčíková, H.; Sheets, H.; Stock, J. H.; Tan, T.; Watson, M.; Wong, T. E.; Anthoff, D., Comprehensive evidence implies a higher social cost of CO2. *Nature* **2022,** *610* (7933), 687-692.

30. Viscusi, W. K., Best Estimate Selection Bias in the Value of a Statistical Life. *Journal of Benefit-Cost Analysis* **2018,** *9* (2), 205-246.

31. Rennert, K.; Prest, B. C.; Pizer, W.; Newell, R. G.; Anthoff, D.; Kingdon, C.; Rennels, L.; Cooke, R.; Raftery, A. E.; Sevcikova, H.; Errickson, F., The Social Cost of Carbon: Advances in Long-Term Probabilistic Projections of Population, GDP, Emissions, and Discount Rates. 2022.

32. Rennert, K.; Prest, B. C.; Pizer, W. A.; Newell, R. G.; Anthoff, D.; Kingdon, C.; Rennels, L.; Cooke, R.; Raftery, A. E.; Sevcikova, H.; Errickson, F., The Social Cost of Carbon: Advances in Long-Term Probabilistic Projections of Population, GDP, Emissions, and Discount Rates. *Brookings Papers on Economic Activity* **2021**, 223-305.

33. Smith, C. J.; Forster, P. M.; Allen, M.; Leach, N.; Millar, R. J.; Passerello, G. A.; Regayre, L. A., FAIR v1.3: a simple emissions-based impulse response and carbon cycle model. *Geosci. Model Dev.* **2018,** *11* (6), 2273-2297.

34. EPA, U. S., Report on the Social Cost of Greenhouse Gases: Estimates Incorporating Recent Scientific Advances. Retrieved from https://www.epa.gov/environmental-economics/scghg. **2023**.

35. Rennert, K.; Prest, B. C.; Pizer, W.; Newell, R. G.; Anthoff, D.; Kingdon, C.; Rennels, L.; Cooke, R.; Raftery, A. E.; Ševčíková, H.; Errickson, F., The Social Cost of Carbon: Advances in Long-Term Probabilistic Projections of Population, GDP, Emissions, and Discount Rates. *Brookings Papers on Economic Activity* **2021**, 223-275.
